# Extensive Healthy Donor Age/Gender Adjustments and Propensity Score Matching Reveal Physiology of Multiple Sclerosis Through Immunophenotyping

**DOI:** 10.3389/fneur.2020.565957

**Published:** 2020-11-27

**Authors:** Paavali A. Hannikainen, Peter Kosa, Christopher Barbour, Bibiana Bielekova

**Affiliations:** Neuroimmunological Diseases Section, Laboratory of Clinical Immunology and Microbiology, National Institute of Allergy and Infectious Diseases, National Institutes of Health, Bethesda, MD, United States

**Keywords:** multiple sclerosis, cerebrospinal fluid, immunophenotyping, age, gender, propensity score matching, flow cytometry

## Abstract

Quantifying cell subpopulations in biological fluids aids in diagnosis and understanding of the mechanisms of injury. Although much has been learned from cerebrospinal fluid (CSF) flow cytometry in neuroimmunological disorders, such as multiple sclerosis (MS), previous studies did not contain enough healthy donors (HD) to derive age- and gender-related normative data and sufficient heterogeneity of other inflammatory neurological disease (OIND) controls to identify MS specific changes.

The goals of this blinded training and validation study of MS patients and embedded controls, representing 1,240 prospectively acquired paired CSF/blood samples from 588 subjects was (1) to define physiological age-/gender-related changes in CSF cells, (2) to define/validate cellular abnormalities in blood and CSF of untreated MS through disease duration (DD) and determine which are MS-specific, and (3) to compare effect(s) of low-efficacy (i.e., interferon-beta [IFN-beta] and glatiramer acetate [GA]) and high-efficacy drugs (i.e., natalizumab, daclizumab, and ocrelizumab) on MS-related cellular abnormalities using propensity score matching.

Physiological gender differences are less pronounced in the CSF compared to blood, and age-related changes suggest decreased immunosurveillance of CNS by activated HLA-DR+T cells associated with natural aging. Results from patient samples support the concept of MS being immunologically single disease evolving in time. Initially, peripherally activated innate and adaptive immune cells migrate into CSF to form MS lesions. With progression, T cells (CD8+ > CD4+), NK cells, and myeloid dendritic cells are depleted from blood as they continue to accumulate, together with B cells, in the CSF and migrate to CNS tissue, forming compartmentalized inflammation. All MS drugs inhibit non-physiological accumulation of immune cells in the CSF. Although low-efficacy drugs tend to normalize it, high-efficacy drugs overshoot some aspects of CSF physiology, suggesting impairment of CNS immunosurveillance. Comparable inhibition of MS-related CSF abnormalities advocates changes within CNS parenchyma responsible for differences in drug efficacy on MS disability progression.

Video summarizing all results may become useful educational tool.

## Introduction

Neuroimmunological diseases of the central nervous system (CNS) are an expanding group of immune-mediated conditions that affect brain and spinal cord. Although the last decade brought exciting advances in antibody (Ab)-based diagnostic testing for some of these conditions (1, 2), the diagnosis of others is based on the combination of clinical suspicion, CNS imaging, and evaluation of cerebrospinal fluid (CSF). Examining the cellular composition of CSF may aid in the diagnostic process. Because cytospin provides limited information and low cellularity in non-infectious neuroimmunological disorders prevents the use of CSF cell blocks, cellular abnormalities in neuroimmunological diseases, including multiple sclerosis (MS) are investigated by flow cytometry. Nevertheless, CSF flow cytometry remains a research test, not performed routinely by clinical laboratories.

In 2014, we published our initial experience (i.e., first 221 subjects) with prospective blood and CSF immunophenotyping of untreated subjects who entered a natural history protocol: “Comprehensive Multimodal Analysis of Patients with Neuroimmunological Diseases of the CNS” (Clinicaltrials.gov identifier NCT00794352) (3). This study identified new and validated previously published changes in immune compositions of blood vs. CSF as well as changes between different neurological diseases.

However, our 2014 study, as well as most MS blood/CSF immunophenotyping studies published thus far, have the following limitations: (1) Although some studies did not even consider effects of age and gender (4, 5), others, including ours, used age and gender as covariates in the statistical models. However, age and gender are also associated with important aspects of MS phenotype: e.g., females are over-represented among MS patients, especially in the relapsing-remitting (RRMS) stage and may have a better prognosis than males. Age is linked to immune senescence and progressive accumulation of disability in MS. Therefore, simple statistical adjustments for age/gender effects may mask changes relevant to MS. To discern disease-specific effects, a more appropriate analysis is to define the effects of gender and age in healthy donors (HD) and then subtract physiological effects from patient data. (2) To facilitate conceptual thinking, previous studies either studied a single MS clinical phenotype (6, 7) or divided MS into traditional RRMS and two progressive (PMS) groups: secondary progressive (SPMS) and primary progressive (PPMS) (8, 9). Instead, analyzing changes across all MS patients in relationship to time (e.g., disease duration [DD]) may support or refute a notion that RRMS and PMS represent different stages of an identical, continuously evolving disease. (3) Studies that investigate the effect of drugs on blood/CSF cellular compositions usually investigate only one drug at a time and rarely include HDs (10–12). This precludes comparison between drugs and determination of whether a drug only inhibits or fully normalizes the studied parameter or, alternatively, exerts effects exceeding physiological ranges. The latter may signify impairment of immune functions and foresee the possibility of adverse events.

Based on the value of sensitive enumeration of immune cells in the CSF to aid the diagnosis of neuroimmunological diseases and guide therapeutic decisions, we continue to prospectively acquire blood/CSF flow cytometry–based immunophenotyping data on all subjects in the NCT00794352 natural history protocol. This paper addresses the aforementioned limitations while also benefitting from recent advances in data analyses, such as propensity score matching.

## Materials and Methods

### Patients and Protocol

This study was conducted at the National Institute of Allergy and Infectious Diseases (NIAID) of the National Institutes of Health (NIH) with approval by the institutional review board. All subjects provided written informed consent and were recruited into “Comprehensive Multimodal Analysis of Patients with Neuroimmunological Diseases of the CNS” (Clinicaltrials.gov identifier NCT00794352). Samples were collected prospectively between February 2011 and August of 2019, representing a total of 1,240 paired samples of blood and CSF collected from 588 individuals.

Before sample collection, all participants underwent a comprehensive diagnostic process, including neurological examination transcribed to iPad-based app NeurEx (13), which automatically calculates Expanded Disability Status Scale score (EDSS; ordinal scale from 0 to 10) (14) and Combinatorial Weight-Adjusted Disability Score (CombiWISE; continuous scale from 0 to 100) (15). All subjects had MRI imaging of the brain and upper cervical spinal cord. Clinical and imaging data were transcribed to a research database, and final diagnosis, together with the rating of diagnostic certainty (as “definite,” “most likely,” and “remains undiagnosed”), was determined and adjusted based on longitudinal follow-up. MS diagnosis was based on 2010 McDonald diagnostic criteria (16) and, after 2017, based on 2017 modifications (17). During longitudinal follow-up, initiation, and termination dates of any immunomodulatory disease-modifying treatment (DMT) were recorded in the database.

Other inflammatory neurological disorders (OIND) include cryptococcal meningitis (33/90 patients), Lyme disease with CNS involvement (10/90), sarcoid (7/90), Susac's syndrome (5/90), neuromyelitis optica (4/90), vasculitis (2/90), Sjogren's syndrome (2/90), and others (27/90). Non-inflammatory disorders (NIND) include Lyme disease with no CNS involvement (43/80), vascular/ischemic disorders (7/80), migraines (6/80), systemic cryptococcosis without CNS involvement (6/80), and others (19/80).

Drug therapy status was adjudicated based on following criteria: (1) To be classified as untreated, a patient was either never treated or had to be off a low-efficacy drug for at least 90 days, off a high-efficacy drug for at least 180 days, and off steroids for at least 30 days. The dichotomization of MS drugs to low vs. high efficacy was based on a published meta-analysis (18). (2) The treated patient had to be on a drug for a minimum of 90 days. Drug effect(s) were analyzed only when the research database contained a minimum of 10 treated patients/drug that fulfilled entry criteria.

### Sample Collection, Processing, and Flow Cytometry

Biological samples were collected between 9 AM and noon; assigned an alphanumeric code that blinded the investigators to the diagnosis, treatment status, and clinical and imaging data; and processed according to standard operating procedures (SOPs). Briefly, ~25 mL of CSF was collected, transported on ice, and processed within 30 min of collection. During processing, CSF cells were transferred from collection tubes into two 15-mL centrifuge tubes (Sarstedt, REF: 62554) and spun for 10 min at 335 g at 4°C. The pelleted CSF cells were combined and resuspended in X-VIVO^TM^ 15 (Lonza, REF: 04-418Q) on ice. For whole blood, the samples were transferred from collection tubes into BD Vacutainer® CPT^TM^ tubes (BD Biosciences, REF: 362753) to isolate mononuclear cells from whole blood. CPT^TM^ tubes were spun according to manufacturer instructions for 30 min at 1,800 g at room temperature, and the cell portion was transferred into 15-mL centrifuge tubes. Blood cells were washed twice in phosphate buffered saline (PBS) (Gibco, REF: 100100-031) and resuspended in X-VIVO^TM^ 15 on ice. CSF cells were concentrated at 100 cells/μl, and cells from blood were concentrated at 2,000 cells/μl. Concentrated cells were counted by staining with trypan blue and using hemocytometer and stored on ice prior to staining.

**Figure 1:**
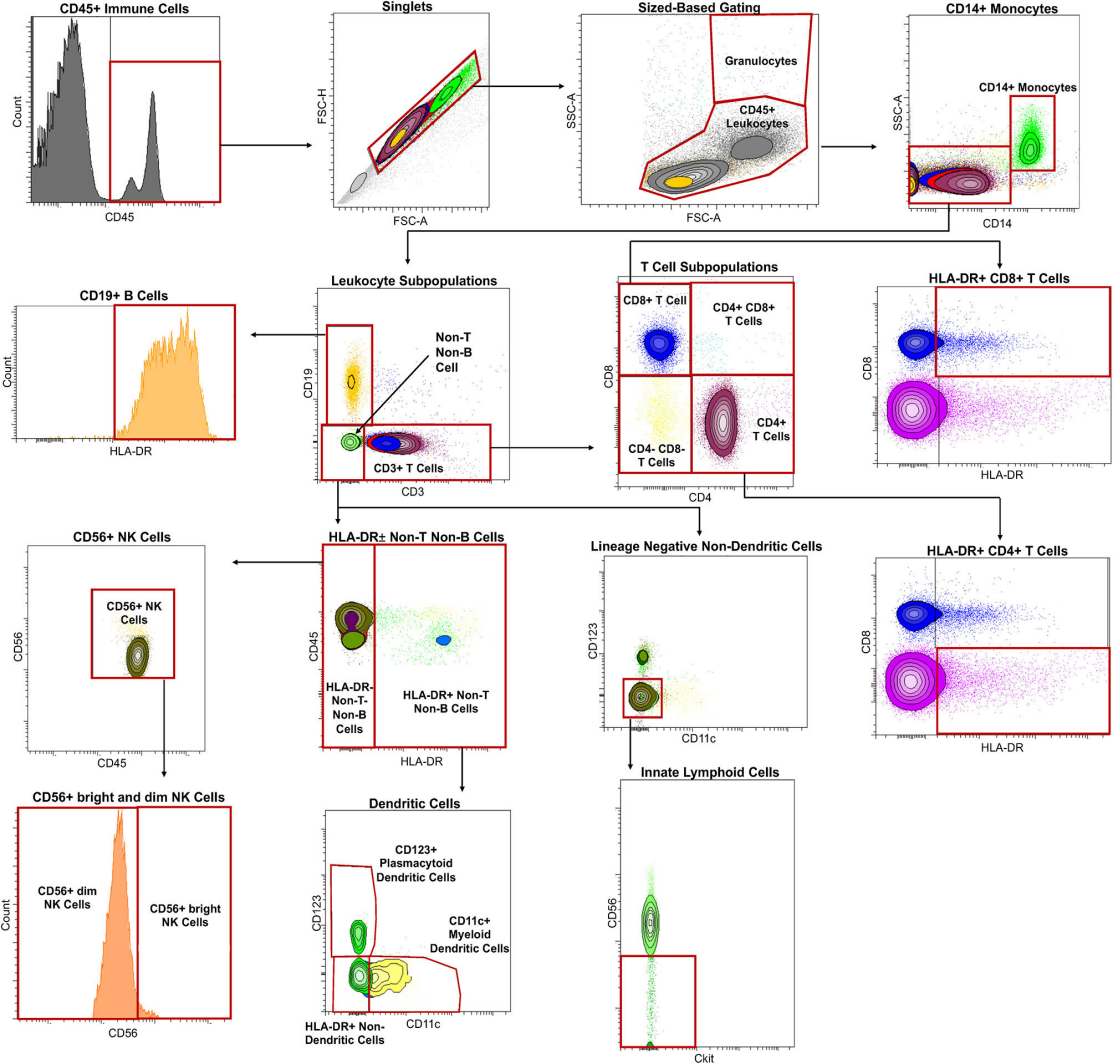
Immune cells were first classified by CD45 expression with histogram gating, followed by gating into singlets with size-based gating [forward scatter area (FSC-A) vs. forward scatter height (FSC-H)]. Granulocytes were distinguished from CD45+ leukocytes with size-based gating as well [FSC-A vs. side scatter area (SSC-A)]. Monocytes were distinguished CD45+ leukocytes using CD14 expression. Leukocyte subpopulations were then identified by subtracting CD14+ monocytes from CD45+ leukocytes and categorized by lack or expression of CD19 or CD3 into adaptive and innate immunity. CD19 expressing cells were confirmed to be CD19+ B cells by gating using of HLA-DR. T cells were identified using CD3 expression with further division into CD4-expressing T cells and CD8-expressing T cells. Activated or HLA-DR-expressing CD4+ T cells and CD8+ T cells were also identified in the same gate by placing HLA-DR in the x-axis and CD8 in the y-axis. Cells lacking CD19 and CD3 expression were categorized into innate immunity and further subdivided by HLA-DR expression. HLA-DR expressing cells as part of innate immunity were divided into dendritic cell populations with myeloid dendritic cells identified using CD11c and plasmacytoid dendritic cells using CD123. HLA-DR negative cells were, on the other hand, identified into NK cell populations by using CD56. CD56+ NK cells were then divided into CD56^dim^ and CD56^bright^ cells using CD56 expression as a guide. Innate lymphoid cells were identified from cells lacking CD3 vs. CD19, CD11c vs. CD123, and Ckit vs. CD56.

A minimum of 2,000 CSF cells and 200,000 blood cells were transferred into a 96-well plate, and Fc receptors on cells were blocked using 2% intravenous human immunoglobulin (Gamunex-C, REF: NDC 13533-800-20) on ice for 5 min. The cells were pelleted for 5 min at 335 g at 4°C and supernatant discarded. A 12-color Ab panel (Supplementary Table 1) was optimized in preliminary experiments to assure saturating concentrations of each antibody. Antibody mixture was added to the pelleted cells and mixed gently with a pipette, then incubated in the dark on ice for 30 min, according to published protocol (3). After staining, cells were washed twice, pelleted by centrifuging at 335 g for 5 min at 4°C, then resuspended in 200 μl of fluorescence-activated cell sorting (FACS) buffer [1 g of sodium azide (Sigma, REF: 26682-2-8), 10 mL of fetal bovine serum (Gemini, REF:100-106), and 1 L of PBS (Gibco, REF: 100100-031)]. After resuspending, the cells were immediately run in a BD Bioscience LSR II flow cytometer with a high-throughput sampler. Cell populations were gated prospectively using BD FACSDiva software based on known distributions of cell surface markers for markers of bimodal distribution and based on isotype control for other markers. The gating strategy is described in Figure 1. Gating was quality controlled weekly and adjusted if gating mistakes were identified before uploading results to the research database and locking them from further modifications. After defining entry criteria for this study, all eligible, prospectively gated results were exported for retrospective analyses.

### Logarithmic Transformation and Outlier Analysis

All analyses was performed in R Studio statistical software (19).

Appropriate cell ratios (number of cells in a population/number of cells in another population), cell proportions (number of cells in a population/number of CD45+ leukocytes), and absolute numbers (number of cells/ml) were calculated and log transformed. Outlier analysis was completed independently in each diagnostic category by excluding any data points in a cell ratio, cell proportion, or absolute number that was outside first quarter (Q1) − 3^*^interquartile range (IRQ) or third quarter (Q3) + 3^*^IQR.

### Age/Gender Adjustments

All cell proportions and absolute numbers were correlated with age separately in CSF, blood, and as CSF/blood ratios using Spearman correlation in the HD cohort. Features that demonstrated significant correlation defined as a *p* ≤ 0.05 adjusted for multiple comparisons using a false discovery rate (FDR) were subsequently adjusted in patient samples using linear regressions derived from the HD cohort: Specifically, the measured values were recalculated as residuals from HD linear regressions as previously described (20).

**Figure 2:**
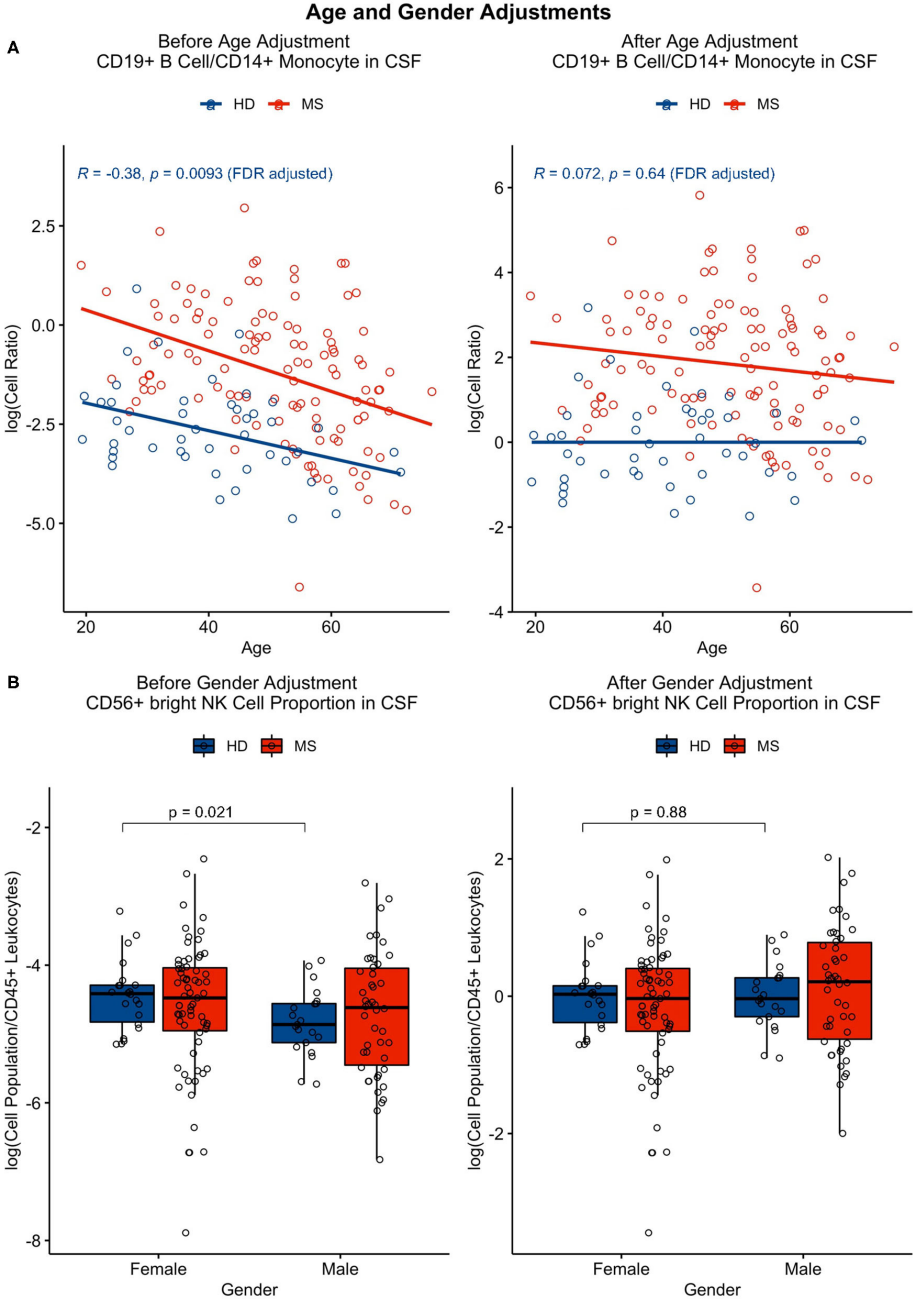
**(A)** Features that correlated significantly (*p* ≤ 0.05) with age in HDs (in blue) were adjusted using linear regression. This adjustment was applied to all patient cohorts, including the MS patient cohort (as shown in red). **(B)** Features that had significant gender differences (*p* ≤ 0.05) were adjusted as well with application of this adjustment to all cohorts by subtracting one gender category as group medians.

The same was performed for analysis of gender differences using an unpaired *t*-test, in which physiological gender effects that remained significant after adjustment for multiple comparisons were subtracted from one gender category as group medians. All features that were found to be affected by age or gender can be found in Tables 1, 2 with examples of adjustments in Figure 2. The R code for age/gender adjustments can be found in GitHub with a link in the Data Availability section.

**Table 1:** Immunophenotyping features were correlated in 48 HDs with age in blood, CSF, and CSF/blood ratios.

**Blood (*****n*** **=** **48)**			**CSF (*****n*** **=** **48)**			**CSF/Blood (*****n*** **=** **48)**		
**Feature**	**Spearman Rho**	***p*-value**	**Feature**	**Spearman Rho**	***p-*value**	**Feature**	**Spearman Rho**	***p-*value**
HLA-DR+ CD4+ T cell/CD45+ leukocyte proportion	0.389	0.007	CD4- CD8- T Cell/CD45+ leukocyte proportion	−0.401	0.007	CD19+ B cell/CD14+ monocyte ratio	−0.440	0.003
CD11c+ myeloid dendritic cell/CD123+ plasmacytoid dendritic cell ratio	0.387	0.007	CD19+ B cell/CD14+ monocyte ratio	−0.385	0.009	Innate lymphoid cell abs	−0.390	0.021
CD123+ plasmacytoid dendritic cell/HLA-DR+ non-T non-B cell ratio	−0.387	0.008	CD19+ B cell/CD45+ leukocyte proportion	−0.355	0.014	HLA-DR+ CD8+ T cell abs	−0.338	0.022
CD4+ T cell abs	0.386	0.009	CD4+ T cell/CD3+ T cell ratio	0.337	0.024	HLA-DR+ CD4+ T cell abs	−0.337	0.024
CD56+ NK cell abs	0.349	0.018						
CD4+ T cell/CD45+ leukocyte ratio	0.336	0.021						
CD56+ dim NK cell abs	0.337	0.023						
HLA-DR+ CD8+ T cell abs	0.319	0.031						
CD3+ T cell/CD45+ leukocyte proportion	0.320	0.035						
CD3+ T cell abs	0.309	0.037						
CD4+ T cell/CD3+ T cell ratio	0.305	0.038						
HLA-DR+ CD4+ T cell abs	0.302	0.038						
CD56+ bright NK cell abs	0.305	0.040						
HLA-DR+ CD8+ T cell/CD8+ cell ratio	0.296	0.044						
CD11c+ myeloid dendritic cell abs	0.292	0.049						

*Correlation coefficient and spearman p-values were calculated after adjustment for multiple comparisons*.

**Table 2:** A *t*-test with adjustment for multiple comparisons was performed in CSF and blood to identify gender differences in immunophenotyping features.

**Blood (*****n*** **=** **48)**				**CSF (*****n*** **=** **48)**			
**Clinical marker**	***p-*value**	**Male mean (*n* = 24)**	**Female mean (*n* = 24)**	**Clinical marker**	***p-*value**	**Male mean (*n* = 24)**	**Female mean (*n* = 24)**
CD4+ T-cell/CD3+ T-cell ratio	0.002	−0.557	−0.425	CD56+ NK cell/CD45+ leukocyte proportion	0.011	−4.190	−3.795
CD4+ T-cell/CD8+ T-cell ratio	0.008	0.614	0.918	CD56+ bright NK cell/CD45+ leukocyte proportion	0.021	−4.827	−4.443
CD19+ B-cell/CD45+ leukocyte proportion	0.010	−2.546	−2.283	CD4+ T-cell/CD45+ leukocyte proportion	0.024	−0.764	−0.620
CD19+ B-cell/CD14+ monocyte ratio	0.011	−0.345	0.202	CD4+ T-cell/CD8+ T-cell ratio	0.041	1.111	1.343
HLA-DR+ CD8+ T-cell/CD45+ leukocyte proportion	0.012	−3.259	−3.918				
CD8+ T-cell/CD3+ T-cell ratio	0.015	−1.171	−1.384				
HLA-DR+ CD4+ T-cell/CD4+ T-cell ratio	0.029	−2.428	−2.786				
CD56+ dim NK cell/CD56+ bright NK cell ratio	0.033	2.948	2.546				
CD4- CD8- cell/CD45+ leukocyte ratio	0.040	−3.128	−3.455				
HLA-DR+ CD8+ T-cell/CD8+ T-cell ratio	0.044	−1.561	−2.001				

*P-values were calculated along with male and female mean values*.

### Training and Validation Cohorts

To find reproducible changes in age-/gender-adjusted flow cytometry parameters between MS patients and HDs, while limiting number of comparisons, we divided untreated MS patients into training and validation cohorts with a 50/50 split, accounting for a comparable distribution of age, gender, disability, and MS subtypes. An unpaired *t*-test analyzed differences between HDs and MS patients in the training cohort first. Significantly different markers (*p* ≤ 0.05, FDR adjusted) from the training cohort were assessed in the validation cohort. Only statistically significant and reproducible features are displayed and discussed here. All validated markers are shown in Table 3. Demographic details of untreated MS patients in training and validation cohorts are described in Supplementary Table 2.

**Table 3:** Features that were statistically different from each other between HDs and RRMS and/or PMS in the training cohort were then validated in an independent validation cohort, and features significant in the validation cohort are described here.

**Blood (*n* = 59)**	**Marker**	***p-*value**	**HD (mean)**	**RRMS (mean)**	**PMS (mean)**	**HD**	**RRMS**	**PMS**
	CD4+ T-cell/CD3+ T-cell ratio	0.003	−0.492	−0.377	−0.352	a	b	b
	CD123+ plasmacytoid dendritic cell/HLA-DR+ non-T non-B cell ratio	0.004	0.000	0.774	0.501	a	b	b
	CD4+ T-cell/CD8+ T-cell ratio	0.005	0.000	0.216	0.417	a	ab	b
	CD3+ T-cell/CD45+ leukocyte proportion	0.012	0.000	−0.044	−0.092	b	ab	a
	CD8+ T-cell/CD3+ T-cell ratio	0.017	0.000	−0.137	−0.269	b	ab	a
	CD56+ dim NK cell abs	0.022	0.000	0.463	−0.271	ab	b	a
	CD56+ NK cell abs	0.022	0.000	0.459	−0.256	ab	b	a
	CD11c+ myeloid dendritic cell abs	0.026	0.000	−0.024	−0.438	b	ab	a
	HLA-DR+ CD4+ T-cell abs	0.036	0.000	0.213	−0.563	b	b	a
	CD56+ bright NK cell abs	0.047	0.000	0.478	−0.208	ab	b	a
	HLA-DR+ CD8+ T-cell abs	0.047	0.000	0.098	−0.618	a	a	b
**CSF (*****n*** **=** **59)**	**Marker**	***p-*****value**	**HD (mean)**	**RRMS (mean)**	**PMS (mean)**	**HD**	**RRMS**	**PMS**
	CD19+ B-cell/CD14 monocyte ratio	3.04E-07	0.000	1.810	1.479	a	b	b
	CD19+ B-cell/CD45+ leukocyte proportion	2.69E-04	0.000	0.881	0.911	a	b	b
	CD56+ NK cell abs	2.69E-04	2.848	4.033	3.716	a	b	b
	CD19+ B-cell abs	4.42E-04	2.235	3.935	3.239	a	b	b
	CD14+ monocyte/CD45+ leukocyte proportion	0.002	−1.976	−2.885	−2.426	b	a	a
	CD56+ bright NK cell abs	0.004	2.100	3.207	2.989	a	b	b
	CD3+ T-cell abs	0.004	6.530	7.539	7.080	a	b	b
	CD4+ T-cell abs	0.005	6.153	7.166	6.747	a	b	b
	CD4+ T-cell/CD45+ leukocyte proportion	0.005	−0.693	−0.447	−0.635	a	b	a
	HLA-DR+ CD8+ T-cell abs	0.017	3.821	4.945	4.349	a	b	ab
	CD3+ T-cell/CD45+ leukocyte proportion	0.019	−0.358	−0.219	−0.286	a	b	ab
	CD8+ T-cell abs	0.021	4.926	5.733	5.448	a	b	ab
	CD11c+ myeloid dendritic cell abs	0.029	3.318	3.957	4.138	a	a	b
	CD123+ plasmacytoid dendritic cell abs	0.039	1.381	2.068	2.332	a	a	b
	CD11c+ myeloid dendritic cell/HLA-DR+ non-T non-B cell ratio	0.041	−0.281	−0.406	−0.390	a	ab	b
	CD56+ dim NK cell/CD45+ leukocyte proportion	0.041	−4.863	−4.546	−4.443	a	ab	b

*For each feature, a p-value adjusted for multiple comparisons is shown, along with mean values of HDs, RRMS, and PMS cohorts, and letters describing which cohorts are significantly different from each other. Cohorts with same letters are insignificantly different from each other in the given feature; meanwhile, cohorts with different letters are significantly different from each other in that feature*.

### Propensity Score Matching to Investigate Effects of MS Therapies

Because our natural history cohort included a limited number of MS patients treated with specific drugs, there was high likelihood of unequal distribution of demographic data between smaller drug-treated cohorts and the large untreated cohort. Thus, we employed propensity score matching, with which each treated MS cohort was matched 1:3 to a highly comparable cohort of untreated MS patients in terms of age, gender, and level of disability. Differences between treated and propensity score–matched untreated MS patients were evaluated using unpaired *t*-tests with adjustment for multiple comparisons. This data was supplemented with longitudinal paired data (i.e., before and after specific therapy for all subjects that had such follow-up CSF in our database) to demonstrate agreement between the propensity score–matched group results and intraindividual before–after treatment results. Due to limited sample sizes, no formal statistics were applied to these before/after comparisons.

**Figure 3:**
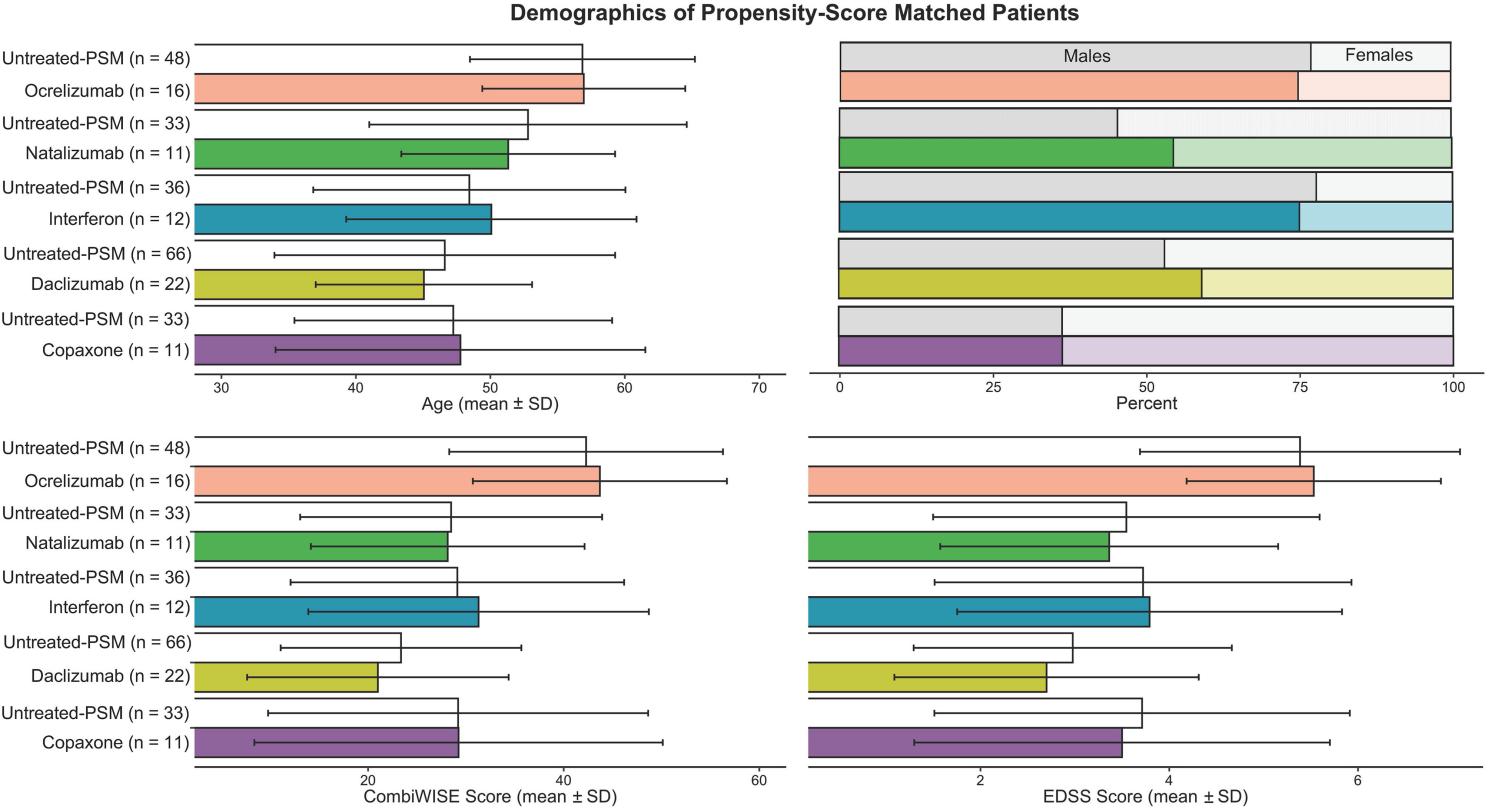
To compare features in untreated and treated MS patients, propensity score matching was performed to allow for appropriate comparisons in features while accounting for differences in age, gender, and disability. Each matched untreated and treated patient group means and ± SD were graphed. For each drug-treated MS patient group, the algorithm picked out from 118 untreated MS patients a cohort of untreated MS patients that is three times larger than the treated group, while also making sure age, gender, and CombiWISE were as close to the treated group as possible.

Figure 3 shows the propensity score–matched age, gender, and disability scales for matched untreated MS and treated MS cohorts. Meanwhile Table 4 describes all patient categories and their demographics

**Table 4:** Demographics of untreated patient cohorts are described here, specifically the number of patients, age, gender, disability, and MS subtype.

	**HD**	**Untreated MS**	**RRMS**	**PPMS**	**SPMS**	**PMS**	**OIND**	**NIND**
Number of patients	48	118	35	46	37	83	90	80
Age (mean ± SD)	40.36 ± 13.37	50.74 ± 12.77	40.35 ± 11.57	54.77 ± 11.55	55.53 ± 9.36	55.11 ± 10.57	45.85 ± 15.65	50.40 ± 14.91
Male/Female	24/24	48/70	12/23	24/22	12/25	36/47	57/33	34/46
CombiWISE (mean ± SD)	7.71 ± 5.06	38.24 ± 19.66	19.47 ± 11.60	46.90 ± 15.69	52.58 ± 12.96	49.00 ± 14.87	N/A	N/A
EDSS (mean ± SD)	0.85 ± 0.86	4.69 ± 2.14	2.43 ± 1.56	5.46 ± 1.73	6.02 ± 1.17	5.70 ± 1.53	N/A	N/A
RRMS/PMS	N/A	35/83	N/A	N/A	N/A	N/A	N/A	N/A

## Results

Because of the quantity of statistically significant results and the complexity of interpretations that integrate paired blood and CSF samples, we developed schematic representations of each results section (Supplementary Presentation 1) and integrated these into a single animated video (Supplementary Video 1).

### Physiological Effects of Age and Gender on Cellular Subpopulations of Blood and CSF

#### Age

Within HDs, we observed (Table 1; Supplementary Presentation 1, Slide 2) the following changes in cellular subpopulations with age in the blood: Both CD3+ T cells and CD4+ T cells correlated positively with age as an absolute number and as a proportion. Multiple HLA-DR+ T cell populations also correlated positively with age, including both HLA-DR+ CD4+ T cell and HLA-DR+ CD8+ T cell absolute numbers and proportions. All NK cell absolute numbers correlated positively with age, including CD56^bright^ and CD56^dim^ NK subsets. Of dendritic cell populations, the absolute number of CD11c+ myeloid dendritic cells (myDC) correlated positively with age along with ratio of CD11c+ myDC to CD123+ plasmacytoid dendritic cells (plDC). CD123+ plDC correlated negatively with age as a ratio to HLA-DR+ non-T non-B cells.

In the CSF, we observed a positive correlation of age with the CD4+ T cell/CD3+ T cell ratio. Both proportions of CD4- CD8- T cells and CD19+ B cells correlated negatively with age in the CSF along with CD19+ B cell/CD14+ monocyte cell ratio.

Several markers as CSF/blood ratios also correlated negatively with age, including both absolute numbers of HLA-DR+ CD4+ T cells and HLA-DR+ CD8+ T cells. The absolute number of innate lymphoid cells and cell ratio of CD19+ B cells/CD14+ monocytes correlate negatively with age.

#### Gender

Within HDs, we observed (Table 2; Supplementary Presentation 1, Slide 3) the following gender-specific differences in cellular subpopulations in the blood: Males had higher proportions of CD4- CD8- T cells, HLA-DR+ CD4+ T cell, HLA-DR+ CD8+ T cells, and CD8+ T cells. Males also had an increased CD56^dim^/CD56^bright^ NK cell ratio. Females, on the other hand, had a higher proportion of CD4+ T cells, CD19+ B cells, CD4/CD8 T cell ratio, and CD19+ B cells/CD14+ monocytes ratio.

In the CSF, the following markers were higher in females compared to males: the proportion of CD4+ T cells, CD4/CD8 T cell ratio, and proportion of both CD56+ NK cells and CD56^bright^ NK cells.

We did not observe any gender differences in CSF/blood ratios.

### Comparison of Cellular Changes Between HDs and Untreated MS Patients

#### RRMS

**Figure 4:**
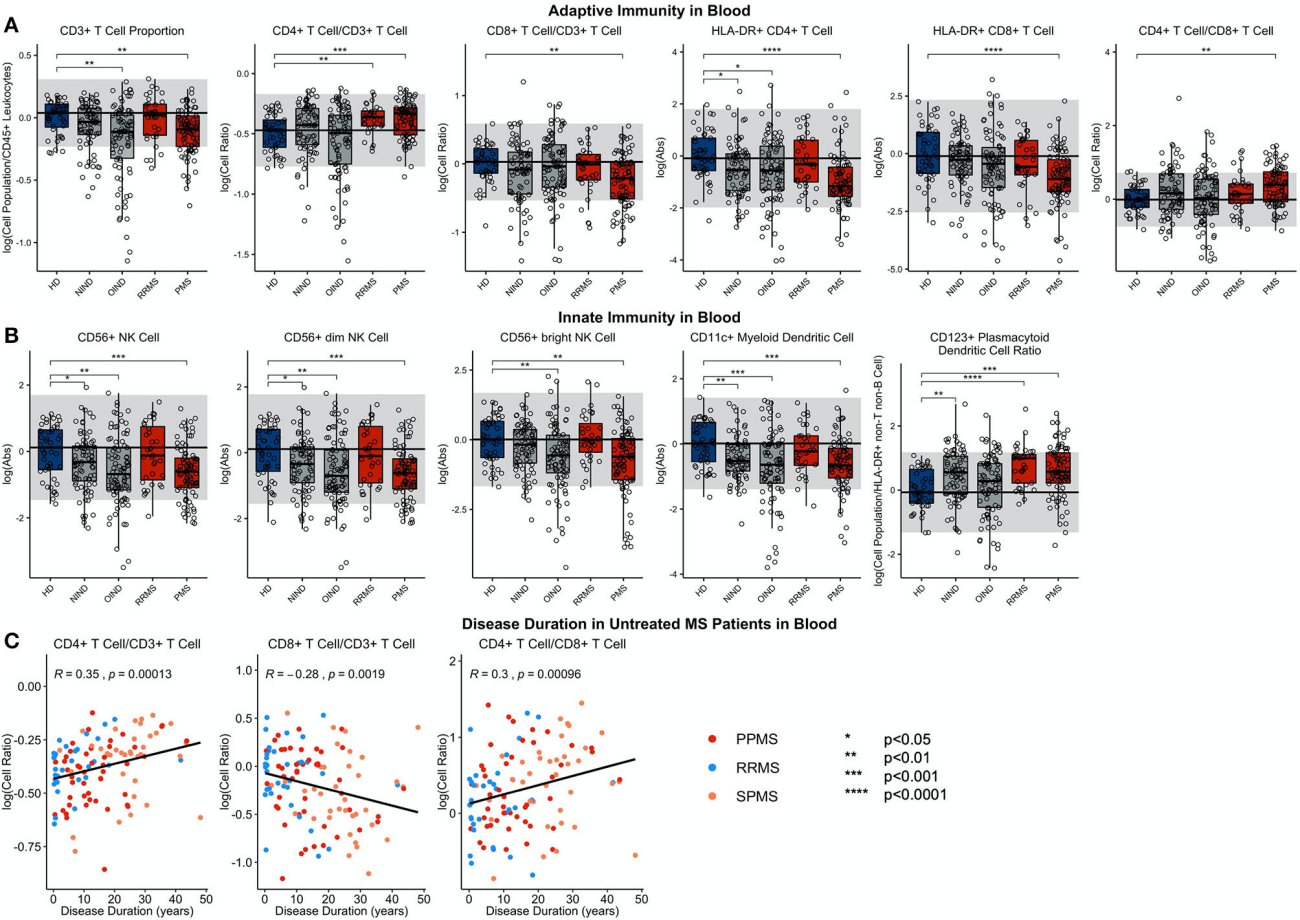
Features that validated in the independent validation cohort in blood during comparison of HDs to RRMS and PMS patients were graphed. HDs, NINDs, OINDs, RRMS, and PMS cohorts for each significant feature were graphed. An unpaired *t*-test was completed to compare each cohort to HDs with adjustment for multiple comparisons. *p < 0.05, **0.01 < *p* < 0.005, ***p < 0.001, ****p < 0.0001. The HD median for each feature was also graphed horizontally and gray shading added representing ± 2 SDs of each feature in HD cohort. **(A)** Features in adaptive immunity. **(B)** Features in innate immunity. **(C)** Features that validated were correlated with disease duration and statistically significant features graphed (*p* ≤ 0.05). MS subtypes were color coded to show heterogeneity of cohorts in each significant feature with blue representing RRMS, red representing PPMS, and orange representing SPMS patients.

In RRMS blood (Figure 4; Supplementary Presentation 1, Slide 4), the proportion of CD4+ T cells (out of all CD3+ T cells) was significantly elevated in comparison to HDs. This increase is MS-specific and cannot be observed in OIND and NIND controls. Of the dendritic cell populations in RRMS, the proportion of CD123+ plDC was increased.

**Figure 5:**
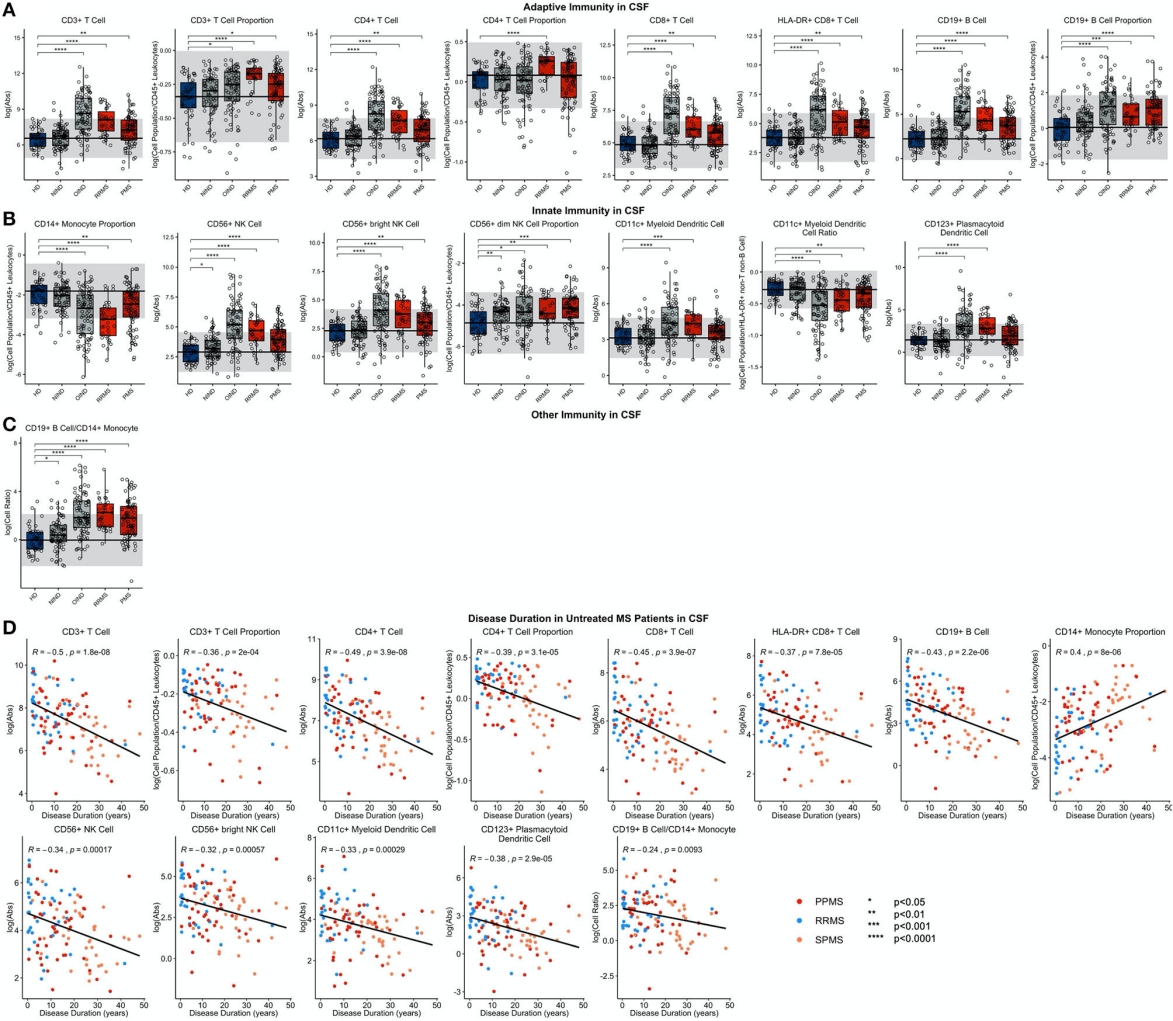
Features that validated in the independent validation cohort in CSF during comparison of HDs to RRMS and PMS patients were graphed. HDs, NINDs, OINDs, RRMS, and PMS cohorts for each significant feature were graphed. An unpaired *t*-test was completed to compare each cohort to HDs with adjustment for multiple comparisons. *p < 0.05, **0.01 < *p* < 0.005, ***p < 0.001, ****p < 0.0001. The HD median for each feature was also graphed horizontally and gray shading added representing ± 2 SDs of each feature in HD cohort. **(A)** Features in adaptive immunity. **(B)** Features in innate immunity. **(C)** Features in other immunity. **(D)** Features that validated were correlated with disease duration and statistically significant features graphed (*p* ≤ 0.05). MS subtypes were color coded to show heterogeneity of cohorts in each significant feature with blue representing RRMS, red representing PPMS, and orange representing SPMS patients.

In RRMS CSF (Figure 5; Supplementary Presentation 1, Slide 4), both the proportion and absolute number of all CD3+ T cells and the CD4+ T cell subset were increased. For CD8+ T cells, only the absolute numbers of CD8+ T cells and their recently activated HLA-DR+ CD8+ T cell subset were increased. The increase in CD4+ T cell proportion was specific to MS. CD19+ B cells were increased as an absolute number and proportion. As reported previously, the proportion of CD14+ monocytes was decreased in the RRMS CSF. The absolute numbers of both NK cell subsets were increased, but proportionally, CD56^dim^ NK cells predominated. Of the dendritic cell populations, the absolute numbers of both DC subsets were increased, but proportionally, CD11c+ myDC were decreased in RRMS compared to HD.

We clearly validated previously reported increase in CSF CD19+ B cell/CD14+ monocyte ratio in RRMS.

Although, to our knowledge, all previous studies separately analyzed differences among diagnostic categories in the blood and CSF, we calculate CSF/blood ratios for each subject, to control CSF levels of immune cells for subject-specific levels of immune cell subpopulations in the blood (Supplementary Figure 1). This allowed us to assess migration of the cells from blood to CSF. CSF/blood ratios of all lymphocyte populations of adaptive immunity were higher in RRMS compared to HD. For innate immunity, CSF/blood ratios of all NK cells, and both subtypes of DCs were likewise increased in RRMS.

#### PMS

Like in RRMS, PMS blood had an increased proportion of CD4+ T cells and increased CD4/CD8 T cell ratio compared to HD (Figure 4; Supplementary Presentation 1, Slide 5). Again, these changes were MS-specific and not observed in other diagnostic groups. However, PMS had decreased blood proportions of all T cells (CD3+), driven by a CD8+ T cells subset. The other important change not observed in RRMS was decrease in the absolute numbers of HLA-DR+ CD4+ T cells and HLA-DR CD8+ T cells.

PMS subjects' blood had also decreased absolute numbers of NK cells—affecting both subtypes—and CD11c+ myDCs. The decrease in myDCs caused a significant proportional increase in CD123+ plDCs.

As we observed in RRMS, PMS CSF had increased absolute numbers of all T cells: CD3+ CD4+, CD8+, and also HLA-DR+ CD8+ (Figure 5; Supplementary Presentation 1, Slide 5). Analogously, CD19+ B cells were increased as an absolute number and proportion.

Also, the innate immune system abnormalities observed in RRMS were reproduced in PMS CSF: a decreased proportion of CD14+ monocytes and increased absolute number of NK cells with the CD56^dim^ subset proportionally dominating. Expectedly, the CD19+ B cell/CD14+ monocyte ratio was increased in PMS CSF.

The similarity of the CSF phenotype between RRMS and PMS patients suggests an identical immune pathophysiology of all MS subtypes. To test this hypothesis, we analyzed correlations of blood and CSF immune populations with MS DD. In the blood, we observed significant positive associations of CD4+ T cell and CD4/CD8 T cell ratios with MS DD (Figure 4); meanwhile, the proportion of CD8+ T cells correlated negatively with DD. Untreated MS patients demonstrated highly statistically significant declines in absolute numbers of all immune cells in the CSF as a function of DD (Figure 5). Only the proportion of monocytes increased with DD. The same conclusions were derived from CSF/blood ratios of immune cells (Supplementary Figure 1). The color-coding of the 3 MS subtypes in all of these correlation plots provides strong evidence for the uniform immune-cell abnormalities in MS that evolve as function of DD and not of clinical classification.

### Drug-Related Effects on MS Inflammation

Having defined blood and CSF cellular abnormalities of untreated MS (with subtracted effects of natural aging and gender), we next investigated the effects of 5 different immunoregulatory treatments of MS using propensity score matching.

#### Interferon-Beta (IFN-Beta)

**Figure 6:**
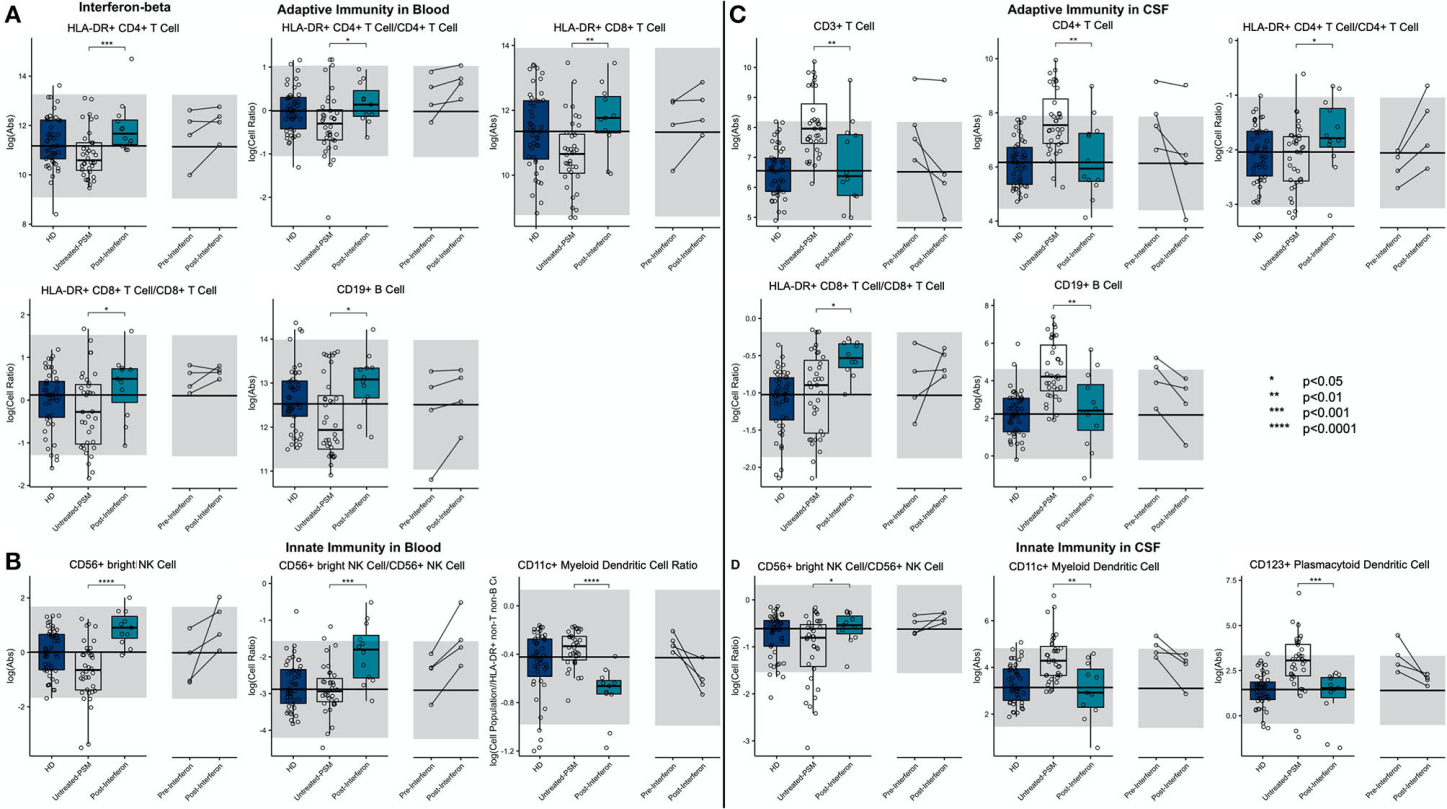
An unpaired *t*-test with adjustment for multiple comparisons was performed for features during comparison of 36 propensity score matched (PSM) untreated MS patients and 12 IFN-beta treated MS patients in blood and CSF. All significant markers were graphed and supplemented with HD cohort and data from 4 longitudinal patients with paired pretreatment and post-treatment data. *p < 0.05, **0.01 < *p* < 0.005, ***p < 0.001, ****p < 0.0001. HD median for each feature was also graphed horizontally and gray shading added representing ± 2 SDs of each feature in HD cohort. **(A)** Features in adaptive immunity in blood. **(B)** Features in innate immunity in blood. **(C)** Features in adaptive immunity in CSF. **(D)** Features in innate immunity in CSF.

IFN-beta treatment led to a highly significant and drug-specific increase in HLA-DR+ T cells, in the blood and CSF. This included all absolute numbers and proportions of HLA-DR+ CD4+ and CD8+ T cells (Figure 6; Supplementary Presentation 1, Slide 6). Also, the CD19+ B cell absolute number was elevated in the blood of IFN-beta-treated patients compared to matched untreated subjects.

We also reproduced a previously published observation that IFN-beta treatment increased the absolute number of CD56^bright^ NK cells and the ratio of CD56^bright^ to CD56+ NK cells. Finally, we saw a highly significant decrease in the proportion of CD11c+ myDCs in the IFN-beta-treated subjects.

Although the absolute number of T cells, especially CD4+ T cells, and CD19+ B cells decreased in the CSF of IFN-beta-treated subjects, the proportions of HLA-DR+ T cells, both CD4+ and CD8+, actually increased, paralleling the same proportional increases of these recently activated T cells in the blood of IFN-beta-treated subjects. In the innate immunity, the proportional increase of CD56^bright^ NK cells propagated from the blood to the CSF of IFN-beta-treated subjects. Additionally, IFN-beta decreased absolute numbers of both subsets of DCs.

The investigation of CSF/blood (Supplementary Figure 2) ratios demonstrates that these IFN-beta-induced changes on immune cell subpopulations (i.e., expansion of HLA-DR+ T cells and CD56^bright^ NK cells), although observed in both compartments, was much greater in the blood than in the CSF.

#### Glatiramer Acetate (GA)

**Figure 7:**
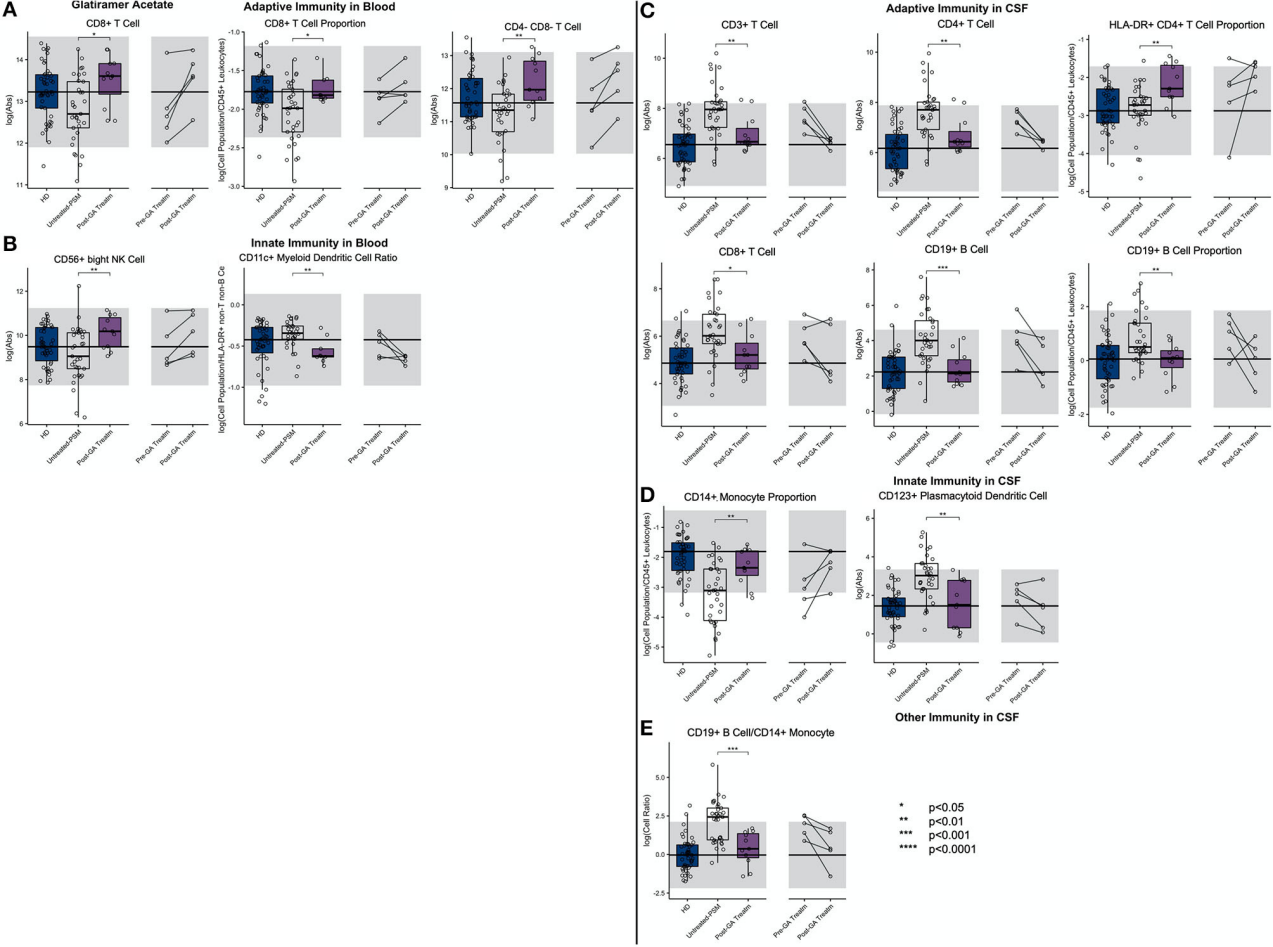
An unpaired *t*-test with adjustment for multiple comparisons was performed for features during comparison of 33 propensity score matched (PSM) untreated MS patients and 11 GA-treated MS patients in blood and CSF. All significant markers were graphed and supplemented with the HD cohort and data from 5 longitudinal patients with paired pretreatment and post-treatment data. *p < 0.05, **0.01 < *p* < 0.005, ***p < 0.001, ****p < 0.0001. The HD median for each feature was also graphed horizontally and gray shading added representing ± 2 SDs of each feature in the HD cohort. **(A)** Features in adaptive immunity in blood. **(B)** Features in innate immunity in blood. **(C)** Features in adaptive immunity in CSF. **(D)** Features in innate immunity in CSF. **(E)** Features in other immunity in CSF.

We observed that, compared to propensity score–matched untreated MS, GA treatment increased absolute numbers and proportions of CD8+ T cells in blood (Figure 7; Supplementary Presentation 1, Slide 7). Surprisingly, GA-treated patients had also higher absolute numbers of immunoregulatory CD56^bright^ NK cells in the blood, and the proportion of CD11c+ myDCs was decreased.

In the CSF of GA-treated MS patients, absolute numbers of all lymphocytes of adaptive immunity (i.e., T and B cells) were significantly decreased compared to untreated MS patients. However, the proportion of HLA-DR+ CD4+ T cells was increased. Within innate immune system cells, GA-treatment elevated CD14+ monocyte proportion in the CSF and normalized B cell/monocyte ratio. GA-treated patients also had normal absolute numbers of plDCs, which were significantly elevated in untreated MS subjects. The examination of CSF/blood ratios (Supplementary Figure 3) only enhanced the aforementioned observations indicating that the prevalent action of GA is overall normalization of MS-associated CSF inflammation.

#### Natalizumab

**Figure 8:**
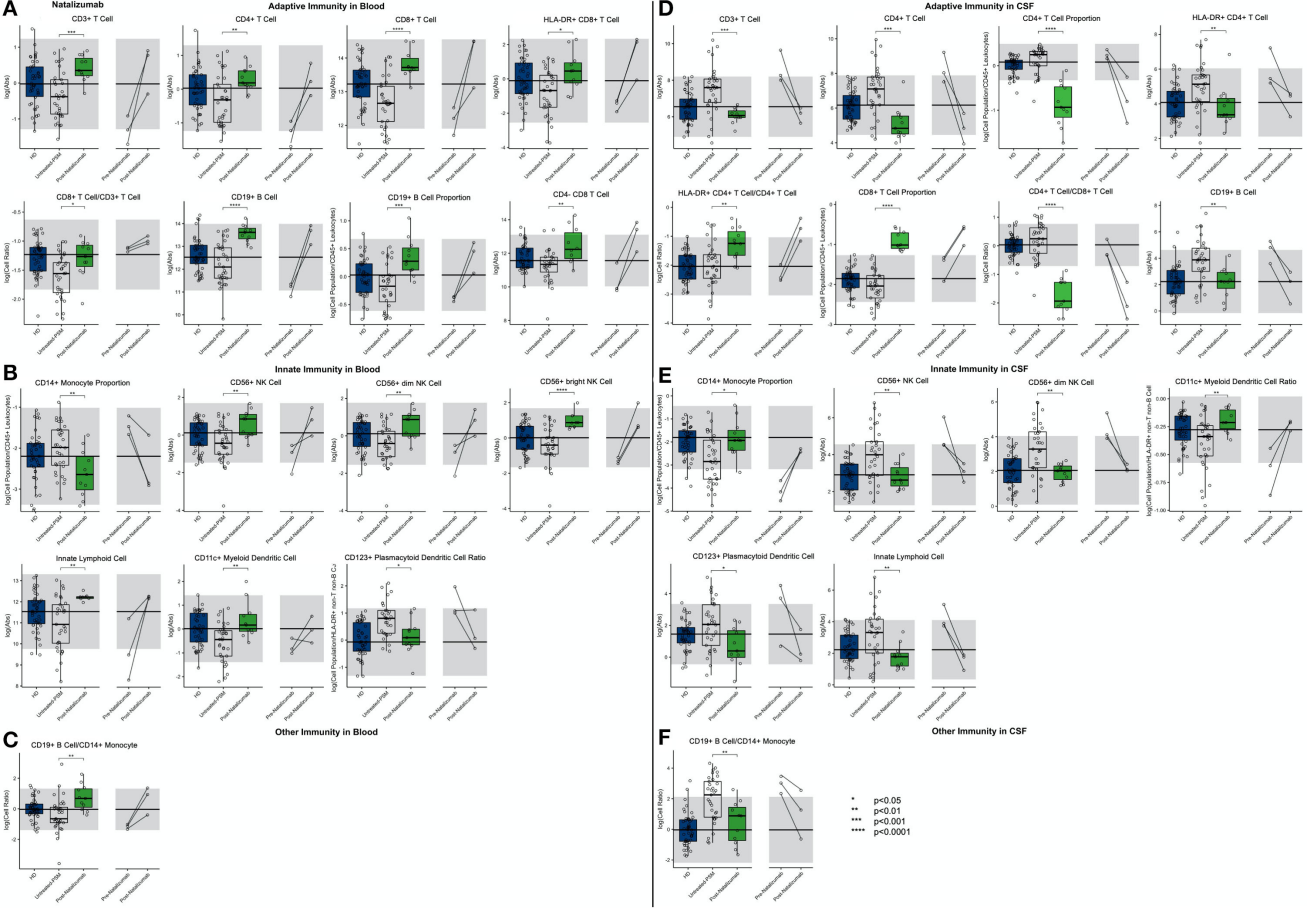
An unpaired *t*-test with adjustment for multiple comparisons was performed for features during comparison of 33 PSM untreated MS patients and 11 natalizumab treated MS patients in blood and CSF. All significant markers were graphed and supplemented with the HD cohort and data from 3 longitudinal patients with paired pretreatment and post-treatment data. *p < 0.05, **0.01 < *p* < 0.005, ***p < 0.001, ****p < 0.0001. The HD median for each feature was also graphed horizontally and gray shading added representing ± 2 SDs of each feature in the HD cohort. **(A)** Features in adaptive immunity in blood. **(B)** Features in innate immunity in blood. **(C)** Features in other immunity in blood. **(D)** Features in adaptive immunity in CSF. **(E)** Features in innate immunity in CSF. **(F)** Features in other immunity in CSF.

The first high-efficacy MS treatment we examined was natalizumab. Consistent with its mechanism of action (MOA) of inhibiting migration of most immune cells (except granulocytes and, to a lesser degree, monocytes) from the blood to the CNS and also to other organs, we reproduced the previously reported increase in most immune cell subpopulations in the blood of natalizumab-treated patients (Figure 8; Supplementary Presentation 1, Slide 8). Not investigated previously, we observed enrichment of innate lymphoid cells (ILCs), NK cells (CD56^bright^ > CD56^dim^), and myDCs in the blood of natalizumab-treated patients while the population of plDCs was proportionally decreased. The stronger inhibition of migration of B cells as compared to monocytes resulted in a strong increase in the B cell/monocyte ratio in the blood of natalizumab-treated subjects.

Reciprocally, all of the aforementioned populations of immune cells were severely depleted from the CSF of natalizumab-treated MS patients, usually significantly below physiological levels. We reproduced stronger inhibitory activity of natalizumab on migration of CD4+ T cells as opposed to CD8+ T cells, resulting in highly non-physiological proportional enrichment of CD8+ T cells over CD4+ T cells in the CSF. On the other hand, the levels of remaining immune cells (i.e., B cells, monocytes, NK cells, and DCs) were normalized in the CSF by natalizumab. Only absolute numbers of ILCs fell below physiological levels upon natalizumab treatment.

Again, examination of CSF/blood ratios (Supplementary Figure 4) only highlighted natalizumab's MOA of inhibiting migration of immune cells from the blood to CSF. The hierarchy of inhibition confirmed a much stronger effect on CD4+ as compared to CD8+ T cells and also highlighted the strong effect on all cells of the innate immunity.

#### Daclizumab

**Figure 9:**
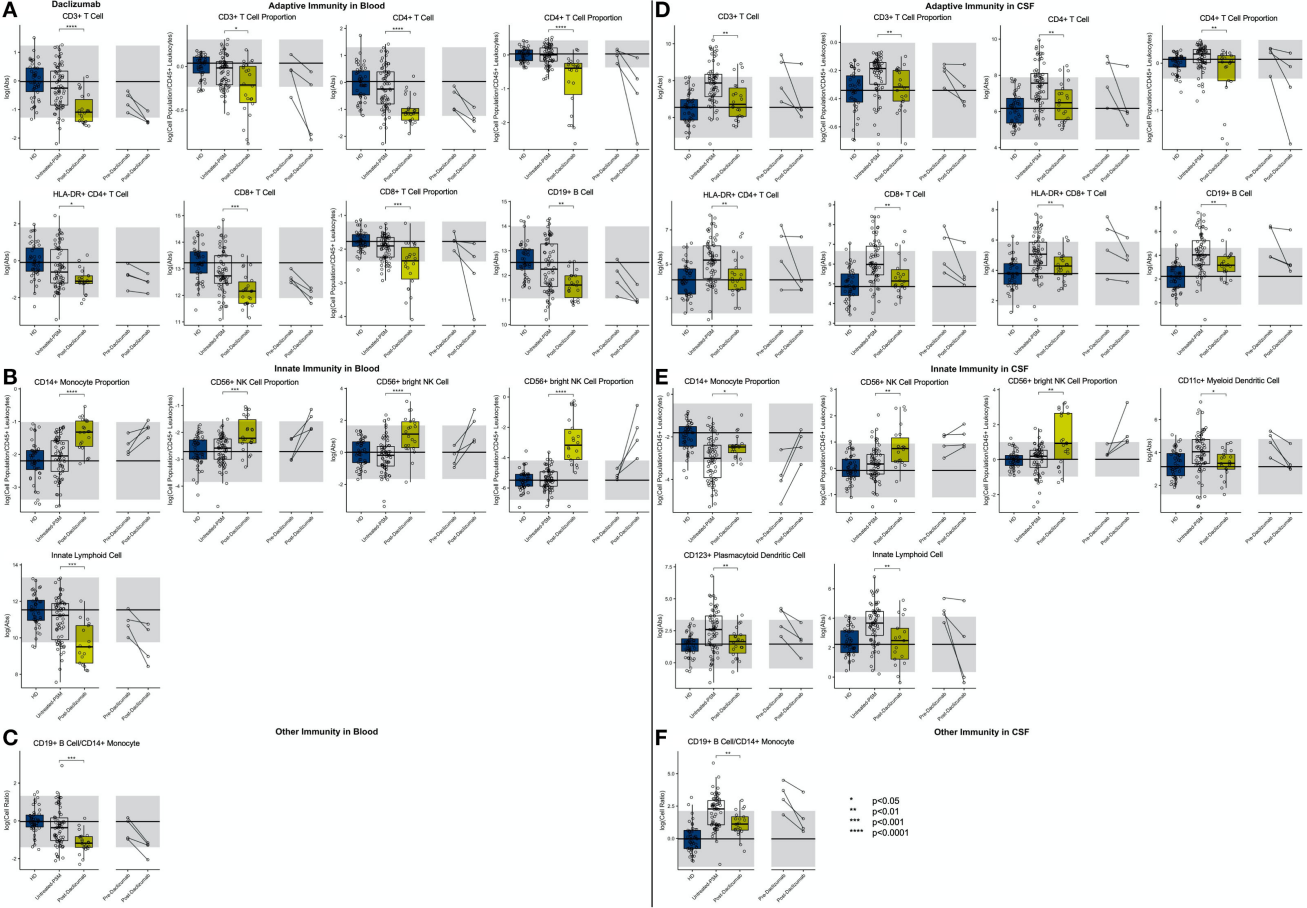
An unpaired *t*-test with adjustment for multiple comparisons was performed for features during comparison of 66 PSM untreated MS patients and 22 daclizumab-treated MS patients in blood and CSF. All significant markers were graphed and supplemented with the HD cohort and data from 4 longitudinal patients with paired pretreatment and post-treatment data. *p < 0.05, **0.01 < *p* < 0.005, ***p < 0.001, ****p < 0.0001. The HD median for each feature was also graphed horizontally and gray shading added representing ± 2 SDs of each feature in the HD cohort. **(A)** Features in adaptive immunity in blood. **(B)** Features in innate immunity in blood. **(C)** Features in other immunity in blood. **(D)** Features in adaptive immunity in CSF. **(E)** Features in innate immunity in CSF. **(F)** Features in other immunity in CSF.

In the blood (Figure 9; Supplementary Presentation 1, Slide 9), daclizumab mildly decreased the absolute numbers and proportions of all lymphocytes of adaptive immunity while significantly expanding CD56^bright^ NK cells and decreasing numbers of ILCs.

In the CSF, daclizumab normalized all MS-associated abnormalities except for proportional expansion of CD56^bright^ NK cells that increased above physiological levels seen in HD. Daclizumab also normalized CSF levels of both DC subsets, and the proportion of monocytes was increased by daclizumab but still did not reach HD levels. Because daclizumab had a stronger inhibitory effect on CSF T cells compared to CSF B cells and failed to completely normalize monocyte numbers, the CSF B cell/monocyte ratio was significantly decreased by daclizumab therapy but remained above HD levels.

CSF/blood ratios (Supplementary Figure 5) showed that, similar to what was observed for IFN-beta, CD56^bright^ NK cell expansion by daclizumab was much higher in the blood than in the CSF, so that the CSF/blood ratio of CD56^bright^ NK cells actually decreased on daclizumab therapy. Finally, we observed that absolute numbers of CSF CD8+ T cells and B cells as well as the B cell/monocyte ratio showed a statistically significant negative correlation with length of treatment, suggesting that the efficacy of daclizumab on these markers of CSF inflammation associated with MS increased with the length of therapy.

#### Ocrelizumab

**Figure 10:**
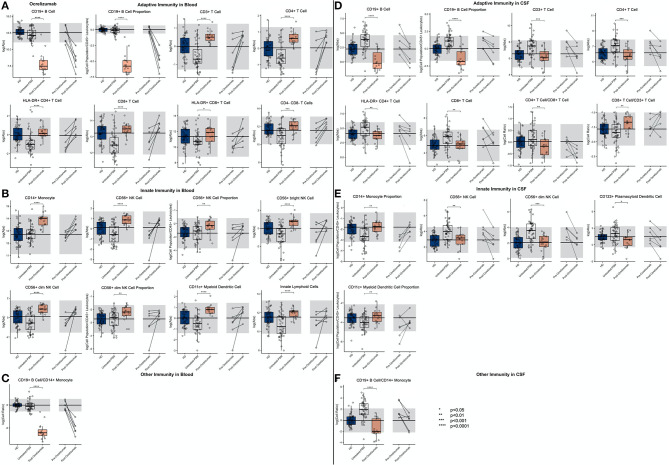
An unpaired *t*-test with adjustment for multiple comparisons was performed for features during comparison of 48 PSM untreated MS patients and 16 ocrelizumab-treated MS patients in blood and CSF. All significant markers were graphed and supplemented with the HD cohort and data from 6 longitudinal patients with paired pretreatment and post-treatment data. *p < 0.05, **0.01 < *p* < 0.005, ***p < 0.001, ****p < 0.0001. The HD median for each feature was also graphed horizontally and gray shading added representing ± 2 SDs of each feature in the HD cohort. **(A)** Features in adaptive immunity in blood. **(B)** Features in innate immunity in blood. **(C)** Features in other immunity in blood. **(D)** Features in adaptive immunity in CSF. **(E)** Features in innate immunity in CSF. **(F)** Features in other immunity in CSF.

As expected, ocrelizumab treatment dramatically depleted blood B cells significantly below physiological levels (Figure 10; Supplementary Presentation 1, Slide 10). Ocrelizumab also exerted milder, nevertheless highly significant effects on other immune cell populations in the blood: increase absolute numbers and/or proportions of most T cell populations and most cells of innate immunity in comparison to propensity score–matched untreated MS subjects. Although most of the paired before–after blood samples demonstrated the same trend, the changes were not congruent in all subjects.

In the CSF, ocrelizumab depleted B cells (again below physiological levels) and normalized most other MS-associated CSF abnormalities. Surprisingly, the inhibitory effect of ocrelizumab was much stronger on CD4+ as compared to CD8+ T cells so that the resulting proportion of CD8+ T cells rose above physiological levels and the CD4/CD8 ratio declined below HD values. MS-associated CSF abnormalities of the innate immunity were normalized by ocrelizumab therapy, including the proportion of monocytes.

**Figure 11:**
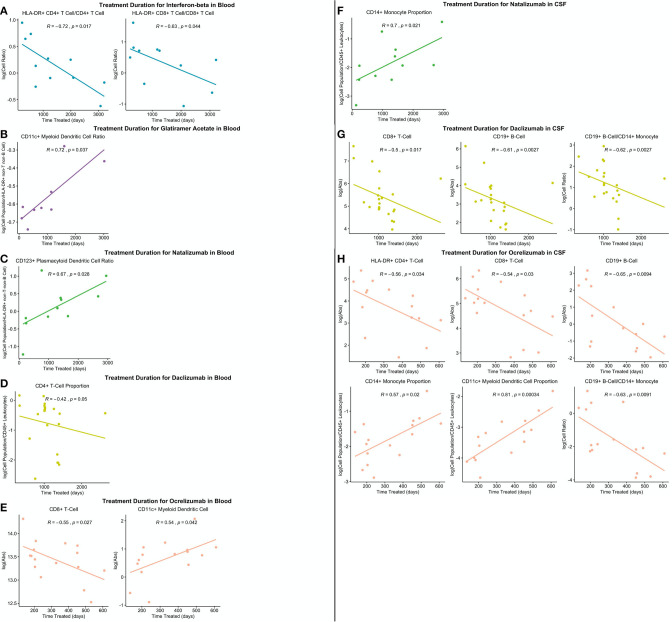
All significantly different features between PSM untreated and treated patients were correlated with treatment duration in treated patients and significant correlations graphed (*p* ≤ 0.05). **(A)** Significant correlations in IFN-beta treated patient blood. **(B)** Significant correlations in GA-treated patient blood. **(C)** Significant correlations in natalizumab-treated patient blood. **(D)** Significant correlations in daclizumab-treated patient blood. **(E)** Significant correlations in ocrelizumab-treated patient blood. **(F)** Significant correlations in natalizumab-treated patient CSF. **(G)** Significant correlations in daclizumab-treated patient CSF. **(H)** Significant correlations in ocrelizumab-treated patient CSF.

Perhaps most importantly, out of all the drugs studied, ocrelizumab exerted the most consistent and broadest treatment duration effects (Figure 11): The absolute number of recently activated HLA-DR+ CD4+ T cells as well as B cells decreased significantly with the treatment duration, and the proportion of CSF monocytes, normally strongly decreased in untreated MS, increased in the CSF of ocrelizumab-treated patients, positively correlating with treatment duration.

Finally, examining CSF/blood ratios (Supplementary Figure 6) showed much stronger B cell depletion in the blood as compared to CSF, and the remaining ocrelizumab-induced changes were more prominent in the CSF, arguing that ocrelizumab may be targeting the essential MS-linked biology.

#### Effect of MS DMTs on Cellular Changes Associated With Physiological Aging

We asked if MS treatments reverse some of the cellular changes associated with physiological aging, depicted in Table 1. To this end, we merged all treatments and used propensity scores to match untreated and treated blood/CSF samples from MS patients (1:1; *n* = 65 for each) and evaluated differences between untreated and treated samples in the age-related features described in Table 1. We saw that, in the blood, MS treatments actually accelerated two cellular changes associated with physiological aging: Treatments increased absolute numbers of CD11c+ myeloid DCs (*p* = 0.04) and CD56+ NK cells (*p* = 0.005). MS DMTs reversed aging-related changes in the CSF as well: they decreased the CD4+/CD3+ T cell ratio (*p* = 0.00084), CD19+ B cell/CD14+ monocyte ratio (*p* = 2.61E-7), and CD19+ B cell/CD45+ leukocyte proportion (*p* = 0.0001). All p-values were adjusted for multiple comparisons.

## Discussion

We start by acknowledging the limitations of current study: (1) Due to its prospective, longitudinal (i.e., spanning >6 years) design, we could not employ recently developed automated flow cytometry analysis techniques (21). Automated algorithms require identical assay parameters, practically achieved by performing only a single or highly limited assay run(s) on cryopreserved samples. This is problematic for CSF applications, as small numbers of CSF cells are poorly amenable to cryopreservation, and cryopreservation affects numbers/phenotype of activated immune cells in unpredictable ways. The obvious advantage of the unsupervised clustering algorithms, limitation of bias, was achieved in this study by use of SOP and strict blinding of the personnel involved in the biological sample processing and running the assay. The data were analyzed weekly, and after quality control, deposited to the research database and locked to prevent further modification. Another advantage of automated clustering is machine learning–aided identification of novel, usually rare cellular populations, possibly related to studied process. We analyzed SOP predefined, rare subpopulations of immune cells (e.g., c-kit+ DCs and ILCs, CD56+ monocytes) using an isotype-guided gating strategy but failed to validate reproducible differences in these rare subpopulations between diagnostic categories. We believe that a key limitation for this effort is small numbers of available CSF cells even when we process >20 cc of CSF and perform immunophenotyping immediately. Because this scarcity of cells would be even more pronounced for cryopreservation-based projects, we consider this limitation insurmountable. Finally, automated methods provide different results based on the algorithm employed (22). Thus, for CSF applications, both automated and manual/blinded approaches have pros and cons and should be viewed as complementary. (2) Our results are based on natural history protocol, with which only the first lumbar puncture (LP) (usually in the untreated stage at the time of diagnostic workup) was a required procedure and the remaining LPs were optional. This led to an uneven representation of treatments captured in this study and a limited number of paired, before/after CSF samples. To mitigate this limitation, we used propensity score matching for formal statistical analyses and provided paired before/after treatment results to assess congruency with group comparisons. The fact that this methodology reproduced a vast majority of published observations of drug-related changes from other investigators (4, 23–34) supports the validity of our approach. (3) Although we used the independent validation cohort to validate immunophenotyping features that differed between MS patients and controls, we had inadequate quantities of biological samples to use the same strategy for HD age/gender effects and to validate treatment-induced changes. Thus, novel findings from these unvalidated experiments require independent validation before they can be considered definite even though all our *p*-values are adjusted for multiple comparisons, and we observed good congruency between propensity score–matched group analyses and paired before–after therapy samples. (4) Some of the analyzed cell subpopulations were enumerated based on “exclusion” gates: e.g., CD4-/CD8- CD3+ T cells or innate lymphoid cells (ILCs). We attempted to identify potentially MS-relevant subpopulations of these “negatively gated” cells using positive markers, such as γδ T cell receptor (TCR) on double-negative T cells and c-kit (FLT3) on ILCs. However, we observed statistically significant differences only in the “parent” negatively gated cells and not in the positively gated subpopulations. Again, we believe that this is caused by the scarcity of these minute cell subpopulations in the CSF even under moderate inflammation associated with MS. Although negatively gated parent cells had to express ubiquitous markers of immune cells CD45 and be localized within a lymphocyte or monocyte forward/side scatter gate, lack of definite positive identification marker of these cells makes us extra cautious in our interpretations. Therefore, we decided to omit these results in the summary models and associated video even though we did include them in all other figures and results.

Notwithstanding these limitations, our results broaden understanding of the evolution of immune cellular changes in blood and CSF compartments across HD and MS lifespans and provide novel, comparative analyses of several MS treatment modalities.

Although we reproduced previously published differences between blood and CSF immune cellular compositions (3, 35–41), to the best of our knowledge, the physiological effects of gender and age on CSF cellular composition have not been previously defined and represent the first novel aspects of this study. Despite the lack of independent validation, our ability to reproduce previously published healthy age/gender effects on *blood* immune cells within an identical HD cohort strengthens the probability that *CSF* data is, likewise, reproducible in future studies. These reproduced age changes in blood include declines in proportion of plDCs with age (42), increases in CD56+ NK cells (43–46) with age, positive correlation of HLA-DR+ CD8+ T cells (47, 48) and HLA-DR+ CD4+ T cells (47) with age, increases in CD4+ T cell/CD3+ T cell ratio with age (49), and increases in proportion of CD3+ T cells (50, 51) and proportion of CD4+ T cells with age (51). Reproduced gender differences in blood include a higher presence of B cells in females (52), higher CD4/CD8 ratio in females (53, 54), higher proportion of CD8+ T cells in males (54), and higher presence of HLA-DR+ CD4+ T cells and HLA-DR+ CD8+ T cells in males (55).

The physiological gender/age effects on cellular composition of the *CSF* were more limited: we observed a higher CD4/CD8 ratio and greater proportion of NK cells in females as compared to male HDs. Physiological aging was associated with lower absolute counts of CSF B cells, higher proportion of CD4+ T cells and, quite surprisingly, lower blood/CSF ratios of HLA-DR+ T cells, both CD4+ and CD8+. This latter change is surprising in view of the higher *blood* levels of activated, HLA-DR+ T cells associated with aging (48) (validated only for HLA-DR+ CD8+ T cells here) and implies decreased migration of these recently activated T cells from blood to CSF. Thus, we conclude that there is likely an age-related decrease in immunosurveillance of CNS tissue, which may, at least partially underlie the epidemiological observations of second peak of CNS malignancies (56, 57) and high morbidity of CNS infections in aged population (58, 59).

After adjusting for physiological gender and age effects and analyzing only samples from untreated subjects, we identified (and validated in the independent cohort) MS-specific changes in the blood: increased proportion of CD4+ T cells (among all T cells) observed in all MS subtypes and an associated increase in CD4/CD8 ratio even though the latter validated statistical significance only in the PMS cohort. Several previous studies found either higher CD4+ T cell counts/proportions in untreated MS (35, 36, 60) or increased proportions of *activated* CD4+ T cells in MS (61–63), using a variety of different activation markers. Although our study cannot address causal relationships, the fact that we observed a statistically significant increase in the proportion of CD4+ T cells and CD4/CD8 ratio with DD in our untreated MS cohort suggest that these changes may be a consequence of the MS disease process. Indeed, a reciprocal association of declining proportion of CD4+ T cells with MS DD was observed in the CSF. There are several interpretations for this reciprocity: (1) decreased recruitment of CD4+ T cells from blood to CSF or (2) increased recruitment of CD8+ T cells from blood to CSF. Additionally, we must remember that the immune cells in the CSF do not equate the cellular composition of CNS tissue once the compartmentalized inflammation was established (64). Indeed, the CSF concentrations of immune cell–specific cell surface markers, such as CD27 (predominantly shed from T cells especially CD8+), CD21 (shed from B cells, especially naive B cells), CD23 (predominantly shed by activated DCs and memory B cells), and CD14/CD163 (predominantly shed by monocytes and macrophages) are equally increased in all MS subtypes (64), indicating similar levels of inflammation in the intrathecal compartment of RRMS and PMS subjects. In fact, the ratio between these shed cell-surface markers and the numbers of the cells of their origin in the CSF represents *in vivo* measure of inflammation compartmentalized to CNS tissue, which demonstrated significantly higher CNS compartmentalization of inflammation in PMS in comparison to RRMS (64). This *in vivo* observation reproduces MS pathology studies (65, 66).

Consequently, a unifying explanation for most observations in the current study is increased recruitment and enhanced retention of immune cells in CNS tissue (i.e., compartmentalized inflammation) as MS evolves. Additional results supporting this unified explanation are (1) in PMS blood, we identified a decrease in absolute numbers and proportions of T cells, affecting all T cell subtypes, including recently activated, HLA-DR+ T cells. This is unexpected, as a proportion of these cells in the blood increases with physiological aging [(48) and this study], and due to the high prevalence of urinary tract infections in PMS patients, one would expect further increase of HLA-DR+ T cells in PMS blood. Because such recently activated effector T cells easily cross the blood–brain barrier, we consider their recruitment to and retention in CNS likely. In agreement, the CSF/blood ratios of HLA-DR+ T cells is significantly increased in both MS subtypes and also in OIND and, for HLA-DR+/CD4+ T cells, weakly but significantly also in NIND controls. This supports the notion that these recently activated T cells are easily recruited to tissues in response to tissue damage. (2) MS patients have increased levels of all measured immune cell subpopulations in the CSF except for monocytes and granulocytes. This inflammatory reaction is not MS specific; subjects with OIND have even higher concentrations of immune cells in the CSF. However, the new finding is that we see statistically significant decreases of all of these CSF cells with MS duration, not as a dichotomized process separating RRMS from PMS (in fact, no immunophenotyping feature reliably differentiated MS subtypes), but as a continuous process. This data strongly supports MS-driven changes that lead to progressive compartmentalization of the inflammatory process to CNS tissue, such as formation of tertiary lymphoid follicles.

Are these cellular changes identified and validated in the untreated MS population normalized by FDA-approved treatments? Based on our inclusion criteria, we had data to answer this question for 5 drugs. Because of the retraction of daclizumab from the markets and previous extensive analyses of the effects of daclizumab on blood and CSF immune cells (25, 31), we used daclizumab only for comparisons and focus the discussion on the remaining four drugs: two low-efficacy drugs classes (i.e., IFN-beta and GA preparations) and two high-efficacy drugs (i.e., natalizumab and ocrelizumab).

All studied drugs decreased levels of adaptive immunity in the CSF, but they did it to different extents: low-efficacy drugs normalized absolute numbers of CSF T cells and CSF B cells, and high-efficacy drugs decreased CSF immune levels below physiological levels: natalizumab decreased absolute levels and proportions of CD4+ but not CD8+ T cells below HD levels while normalizing CSF B cells, monocytes and NK cells. The relative selectivity of natalizumab's action to CD4+ T cells led to a non-physiological decrease in CSF CD4/CD8 ratio and highly non-physiological increase in the proportion of CSF CD8+ T cells. Ocrelizumab predictably decreased CSF B cells below physiological levels and normalized the remaining CSF immunophenotyping parameters except for the CD4/CD8 T cell ratio, which decreased below physiological levels due to the relative increase in the proportion of CD8+ T cells. Thus, the studied low-efficacy drugs normalized MS-associated changes in CSF lymphocytes, whereas high-efficacy treatments introduced non-physiological changes with more pronounced decrease in CD4+ T cells as compared to CD8+ T cells and non-physiological decreases in CSF CD4/CD8 ratio. This was not observed with daclizumab, which decreased CSF levels of CD4+ and CD8+ T cells to a similar extent.

The other important differences between drug effects on CSF cellular populations resided in their effects on HLA-DR+ T cells: in general, low-efficacy drugs failed to inhibit these recently activated T cells. In fact, IFN-beta significantly increased the proportion of both CD4+ and CD8+/HLA-DR+ T cells, and GA increased the proportion of CD4+/HLA-DR+ T cells. The effect of IFN-beta on these cells is unique as it also increased both absolute numbers and proportions of HLA-DR+ CD4+ and CD8+ T cells in the blood, arguing for a drug-specific effect that may not be beneficial for MS. Actually, our human observations agree with the anti-apoptotic effect on activated T cells of type-1 interferons described in animals (23). This suggests that IFN-beta may have both beneficial effects for MS, such as stabilization of the blood–brain barrier and increasing proportions of regulatory CD56^bright^ NK cells (30, 32) while its unwanted effects include persistence of activated T cells, which may enhance antiviral immunity but may be detrimental in MS.

Natalizumab also failed to normalize HLA-DR+ T cells: the proportion of CD4+/HLA-DR+ T cells increased in the CSF of natalizumab-treated patients due to either lower inhibition of their migration from blood or their subsequent expansion in the lymphocyte-depleted intrathecal compartment. Only ocrelizumab (and daclizumab) normalized CSF numbers of HLA-DR+ T cells. Perhaps most importantly, we observed significant correlations between the reversal of CSF immunophenotyping abnormalities and duration of treatment for ocrelizumab (and, to a lesser degree, for daclizumab). Other tested drugs did not exhibit significant relationships between treatment duration and normalization of MS-related immunophenotyping abnormalities. This suggests that the efficacy of ocrelizumab increases with treatment duration, at least for 2 years, which was the longest CSF follow-up in our cohort. This result is important for the long-term use of ocrelizumab in MS.

When it comes to cells of innate immunity, low-efficacy drugs normalized CSF levels of myDC (IFN-beta only) and plDC (both drugs) but failed to normalize CSF levels of NK cells and monocytes. GA exerted beneficial and statistically significant effects on monocytes, but median levels of CSF monocytes in the treated group were still below the HD median. IFN-beta additionally increased levels/proportions of immunoregulatory CD56^bright^ NK cells, in both blood and CSF, consistent with published studies (30, 32). The high-efficacy drugs had almost identical effects on CSF innate immunity: both normalized numbers of NK cells in the CSF (especially cytotoxic CD56^dim^ NK cells) and increased proportion of monocytes. They also decreased numbers of CSF plDCs but had no significant effects on myDCs. Thus, three MS drugs exerted higher inhibitory effect on plDCs as compared to myDCs (i.e., GA, natalizumab, and ocrelizumab), whereas IFN-beta preparations and daclizumab inhibited both DC subsets equally. Interestingly, these two drugs also significantly expanded CD56^bright^ NK cells in both blood and CSF even though daclizumab exerted stronger effects. It would be interesting to explore the functional relationships between CD56^bright^ NK cells and myDCs in future MS studies.

The analysis of the drug's effects on cell populations in the blood and on blood/CSF ratios provide additional insight into the MOA of these drugs. GA increased blood levels of CD8+ T cells, CD4-/CD8- T cells, and CD56^bright^ NK cells. All these cell types were decreased in untreated MS, especially PMS, in comparison to HDs. GA also normalized CSF/blood ratios for all cells of adaptive immunity. Thus, we interpret these changes as normalization of MS-associated disturbances in blood immune cells. In contrast, IFN-beta exerted several non-physiological blood changes. In addition to previously mentioned increases in HLA-DR+ CD4+ and CD8+ T cells and CD56^bright^ NK cells, IFN-beta preparations also increased the number of blood B cells above physiological levels and significantly decreased the proportion of blood myDCs. These changes are consistent with known direct and indirect activating effects of type-1 interferons on immune cells: e.g., the stimulation of proliferation of NK cells and T cells is IL-15 driven (34), explaining preferential expansion of CD56^bright^ NK cells and HLA-DR+ T cells that express high levels of CD122 and CD132, which together constitute intermediate-affinity IL-2 receptor used for IL-15 trans-presentation.

By inhibiting the migration of immune cells from blood to tissues, natalizumab induces non-physiological increases of blood numbers of most cells of adaptive immunity and both types of NK cells even though the effect on CD56^bright^ NK cells is more robust in comparison to CD56^dim^ NK cells. In contrast, migration of cells of myeloid lineage, such as monocytes and granulocytes, is not significantly inhibited by natalizumab, and their blood numbers are not affected. The highly non-physiological effects of natalizumab on CSF/blood ratios, not shared by the remaining drugs studied in this paper, reflects its known MOA: inhibiting cell migration from blood to tissues with stronger inhibition of CD4+ T cell in comparison to CD8+ T cells, leading to strong proportional enrichment of CD4+ T cells in the blood and CD8+ T cells in the CSF of treated patients (4). The significant effects on CSF/blood ratios of both types of NK cells, both types of DCs, and ILC support the inhibitory action of natalizumab on extravasation of these innate immune cells CSF. As expected, ocrelizumab caused selective non-physiological depletion of blood B cells. This was associated with normalization and/or even overshoot of MS-associated disturbances in cellular blood composition, such as increase in absolute numbers of blood T cells, both CD4+ and CD8+ and their HLA-DR+ subpopulations, but also blood monocytes and NK cells (CD56^bright^>CD56^dim^). Because the absolute numbers of most of these cells increased in the blood and simultaneously decreased in the CSF, CSF/blood ratios mostly decreased below physiological levels. The striking exceptions were CSF/blood ratios of B cells. B cells were depleted in both compartments, but proportionally more in the blood, causing paradoxical increase in CSF/blood B cell ratio above the untreated matched MS patients.

In conclusion, our results support the notion that MS is a single disease with clinical categories of RRMS, SPMS, and PPMS representing different stages of a continuous disease evolution. From the standpoint of cellular immunity, the MS evolution is characterized by constant recruitment of activated T cells to CNS tissue and establishment of an environment conducive to compartmentalized inflammation. All studied MS drugs inhibit cellular inflammatory CSF responses in comparison to untreated MS. From all studied drugs, GA induces least non-physiological changes in cellular immunity while achieving comparable if not better inhibition of CSF inflammation in comparison to IFN-beta preparations. IFN-beta have a disadvantage of inducing chronic activation of the immune system. High-efficacy drugs exert stronger anti-inflammatory effects in CSF but induce many non-physiological changes that may underlie associated risk of infectious complications or cancers under long-term use. Natalizumab is clearly worse in this regard than ocrelizumab. The efficacy of ocrelizumab on the CSF inflammatory response increases with the length of treatment, at least for 2 years. The difference between high- and low-efficacy drugs on disability progression likely reflects more potent inhibition of inflammation in CNS tissue even though this could not be addressed in the current study.

The associated video that integrates the major results of this study may be used for education.

## Data Availability Statement

The datasets presented in this study can be found in online repositories. The names of the repository/repositories and accession number(s) can be found at: https://github.com/vanessatmorgan/Bielekova-Lab-Code/tree/master/FormerLabMembers/Paav.

## Ethics Statement

The studies involving human participants were reviewed and approved by National Institutes of Health (NIH) Central Institutional Review Board (IRB). The patients/participants provided their written informed consent to participate in this study.

## Author Contributions

BB designed the study and guided/supervised all its aspects. PH contributed to data collection and analyzed the data. PK contributed to quality control and maintenance of data and contributed to data analysis. CB exported data and contributed to data analysis. BB and PH wrote the manuscript. All authors contributed to manuscript revision, read, and approved the submitted version.

## Conflict of Interest

BB is co-inventor on several NIH patents related to daclizumab therapy for MS, and as such, has received patent royalty payments from NIH. The remaining authors declare that the research was conducted in the absence of any commercial or financial relationships that could be construed as a potential conflict of interest.

## Supplementary Material

Supplementary Figure 1 Features that are validated in the independent validation cohort in CSF and/or blood were calculated as CSF/blood ratios. CSF/blood ratios were then compared between HDs and RRMS and PMS patients, and statistically significant features graphed. HDs, NINDs, OINDs, RRMS, and PMS cohorts for each feature were graphed. An unpaired *t*-test was completed to compare each cohort to HDs with adjustment for multiple comparisons. ^*^*p* < 0.05, ^**^0.01 < *p* < 0.005, ^***^*p* < 0.001, ^****^*p* < 0.0001. The HD median for each feature was also graphed horizontally and gray shading added representing ± 2 SDs of each feature in the HD cohort. **(A)** Features in adaptive immunity. **(B)** Features in innate immunity. **(C)** Features in other immunity. **(D)** Features that validated were correlated with DD and statistically significant features graphed (*p* ≤ 0.05). MS subtypes were color-coded to show heterogeneity of cohorts in each significant feature with blue representing RRMS, red representing PPMS, and orange representing SPMS patients.

Supplementary Figure 2 Features that were statistically significant in blood and/or CSF between PSM untreated and IFN-beta treated MS patients were calculated as CSF/blood ratios. CSF/blood ratios were then compared between 36 PSM untreated and 12 IFN-beta treated MS patients and an unpaired *t*-test with adjustment for multiple comparisons performed. All significant markers were graphed and supplemented with the HD cohort and data from 4 longitudinal patients with paired pretreatment and post-treatment data. ^*^*p* < 0.05, ^**^0.01 < *p* < 0.005, ^***^*p* < 0.001, ^****^*p* < 0.0001. The HD median for each feature was also graphed horizontally and gray shading added representing ± 2 SDs of each feature in the HD cohort. **(A)** Features in adaptive immunity. **(B)** Features in innate immunity.

Supplementary Figure 3 Features that were statistically significant in blood and/or CSF between PSM untreated and GA-treated MS patients were calculated as CSF/blood ratios. CSF/blood ratios were then compared between 33 PSM untreated and 11 GA-treated MS patients and an unpaired *t*-test with adjustment for multiple comparisons performed. All significant markers were graphed and supplemented with HD cohort and data from 5 longitudinal patients with paired pretreatment and post-treatment data. ^*^*p* < 0.05, ^**^0.01 < *p* < 0.005, ^***^*p* < 0.001, ^****^*p* < 0.0001. The HD median for each feature was also graphed horizontally and gray shading added representing ± 2 SDs of each feature in the HD cohort. **(A)** Features in adaptive immunity. **(B)** Features in innate immunity. **(C)** Features in other immunity.

Supplementary Figure 4 Features that were statistically significant in blood and/or CSF between PSM untreated and natalizumab-treated MS patients were calculated as CSF/blood ratios. CSF/blood ratios were then compared between 33 PSM untreated and 11 natalizumab-treated MS patients and an unpaired *t*-test with adjustment for multiple comparisons performed. All significant markers were graphed and supplemented with HD cohort and data from 3 longitudinal patients with paired pretreatment and post-treatment data. ^*^*p* < 0.05, ^**^0.01 < *p* < 0.005, ^***^*p* < 0.001, ^****^*p* < 0.0001. The HD median for each feature was also graphed horizontally and gray shading added representing ± 2 SDs of each feature in the HD cohort. **(A)** Features in adaptive immunity. **(B)** Features in innate immunity. **(C)** Features in other immunity.

Supplementary Figure 5 Features that were statistically significant in blood and/or CSF between PSM untreated and daclizumab-treated MS patients were calculated as CSF/blood ratios. CSF/blood ratios were then compared between 66 PSM untreated and 22 daclizumab-treated MS patients and an unpaired *t*-test with adjustment for multiple comparisons performed. All significant markers were graphed and supplemented with HD cohort and data from 4 longitudinal patients with paired pretreatment and post-treatment data. ^*^*p* < 0.05, ^**^0.01 < *p* < 0.005, ^***^*p* < 0.001, ^****^*p* < 0.0001. The HD median for each feature was also graphed horizontally and gray shading added representing ± 2 SDs of each feature in the HD cohort. **(A)** Features in innate immunity.

Supplementary Figure 6 Features that were statistically significant in blood and/or CSF between PSM untreated and ocrelizumab-treated MS patients were calculated as CSF/blood ratios. CSF/blood ratios were then compared between 48 PSM untreated and 16 ocrelizumab-treated MS patients and an unpaired *t*-test with adjustment for multiple comparisons performed. All significant markers were graphed and supplemented with HD cohort and data from 6 longitudinal patients with paired pretreatment and post-treatment data. ^*^*p* < 0.05, ^**^0.01 < *p* < 0.005, ^***^*p* < 0.001, ^****^*p* < 0.0001. The HD median for each feature was also graphed horizontally and gray shading added representing ± 2 SDs of each feature in HD cohort. **(A)** Features in adaptive immunity. **(B)** Features in innate immunity. **(C)** Features in other immunity.

Supplementary Presentation 1 Major results of this study are described in this presentation, beginning with a description of immune cell populations in healthy blood and CSF and further narration of age and gender effects on these populations in HDs. Then RRMS and PMS patient blood and CSF immune system physiologies are differentiated from HDs. Last, IFN-beta, GA, natalizumab, daclizumab, and ocrelizumab treated MS patients are compared to untreated MS patients in this 15-min video.

Supplementary Video 1 Major results of this study are described in this educational video beginning with a description of immune cell populations in healthy blood and CSF and further narration of age and gender effects on these populations in HDs. Then RRMS and PMS patient blood and CSF immune system physiologies are differentiated from HDs. Last, IFN-beta, GA, natalizumab, daclizumab, and ocrelizumab treated MS patients are compared to untreated MS patients in this 15-min video.
