# Supplementary figures and images for: Extensive Healthy Donor Age/Gender Adjustments and Propensity Score Matching Reveal Physiology of Multiple Sclerosis Through Immunophenotyping
Source: Front Neurol. 2020 Nov 27;11:565957. doi: 10.3389/fneur.2020.565957 (PMC7732581; doi:10.3389/fneur.2020.565957)

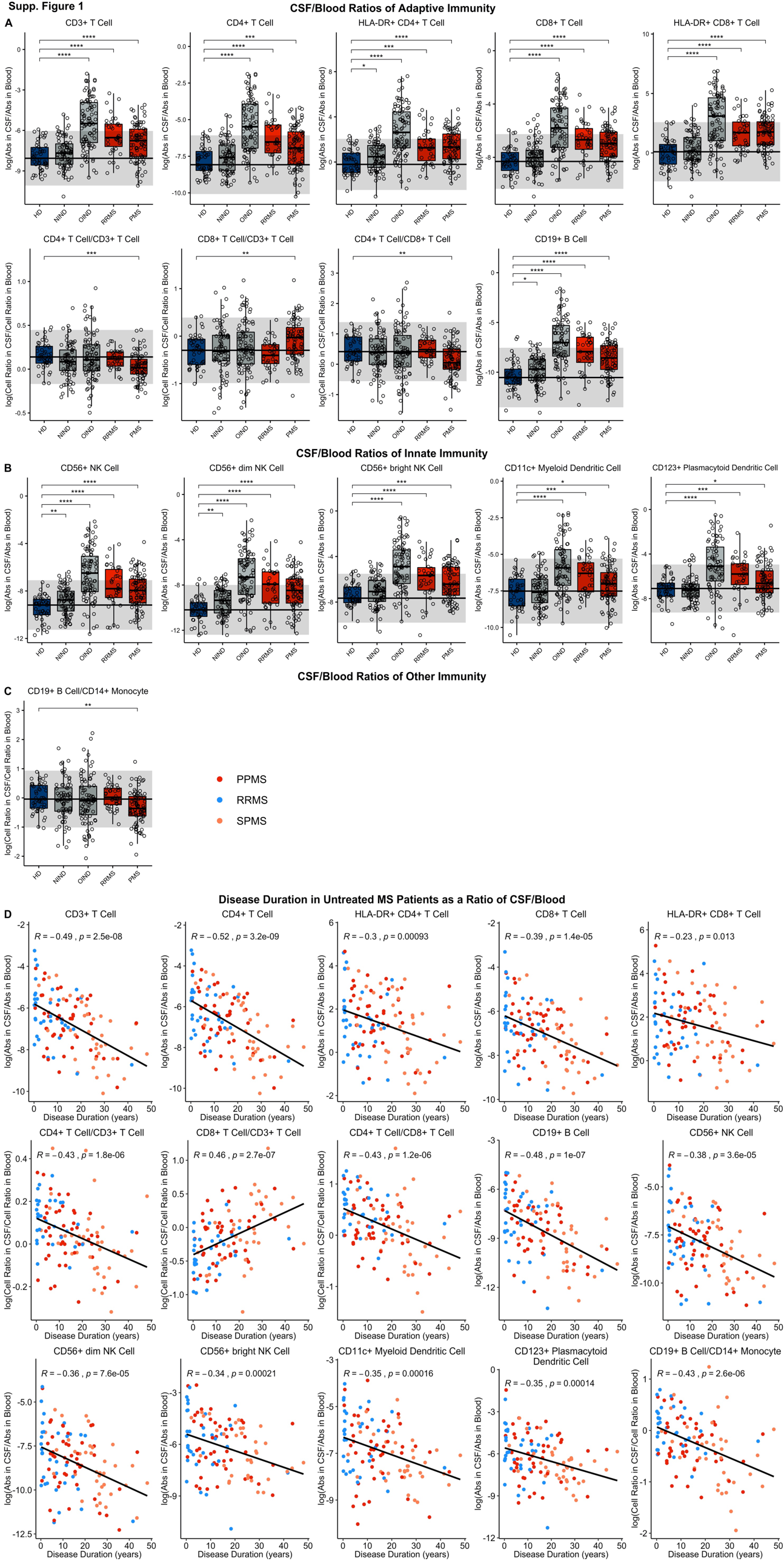

Supplement: Supplementary Figure 1 — Features that are validated in the independent validation cohort in CSF and/or blood were calculated as CSF/blood ratios. CSF/blood ratios were then compared between HDs and RRMS and PMS patients, and statistically significant features graphed. HDs, NINDs, OINDs, RRMS, and PMS cohorts for each feature were graphed. An unpaired t-test was completed to compare each cohort to HDs with adjustment for multiple comparisons. *p < 0.05, **0.01 < p < 0.005, ***p < 0.001, ****p < 0.0001. The HD median for each feature was also graphed horizontally and gray shading added representing ± 2 SDs of each feature in the HD cohort. (A) Features in adaptive immunity. (B) Features in innate immunity. (C) Features in other immunity. (D) Features that validated were correlated with DD and statistically significant features graphed (p ≤ 0.05). MS subtypes were color-coded to show heterogeneity of cohorts in each significant feature with blue representing RRMS, red representing PPMS, and orange representing SPMS patients. [file Image_1.tif]

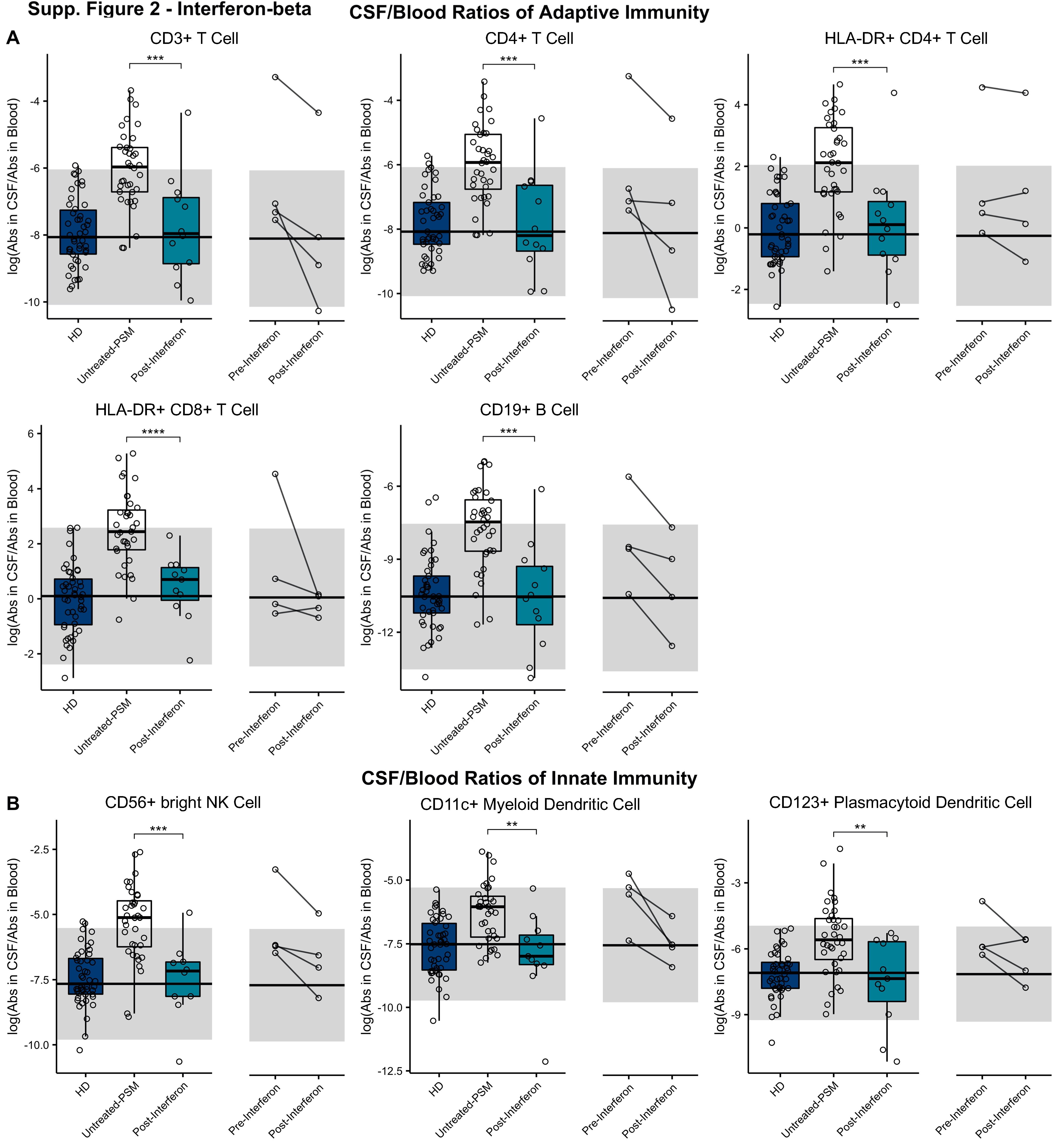

Supplement: Supplementary Figure 2 — Features that were statistically significant in blood and/or CSF between PSM untreated and IFN-beta treated MS patients were calculated as CSF/blood ratios. CSF/blood ratios were then compared between 36 PSM untreated and 12 IFN-beta treated MS patients and an unpaired t-test with adjustment for multiple comparisons performed. All significant markers were graphed and supplemented with the HD cohort and data from 4 longitudinal patients with paired pretreatment and post-treatment data. *p < 0.05, **0.01 < p < 0.005, ***p < 0.001, ****p < 0.0001. The HD median for each feature was also graphed horizontally and gray shading added representing ± 2 SDs of each feature in the HD cohort. (A) Features in adaptive immunity. (B) Features in innate immunity. [file Image_2.tif]

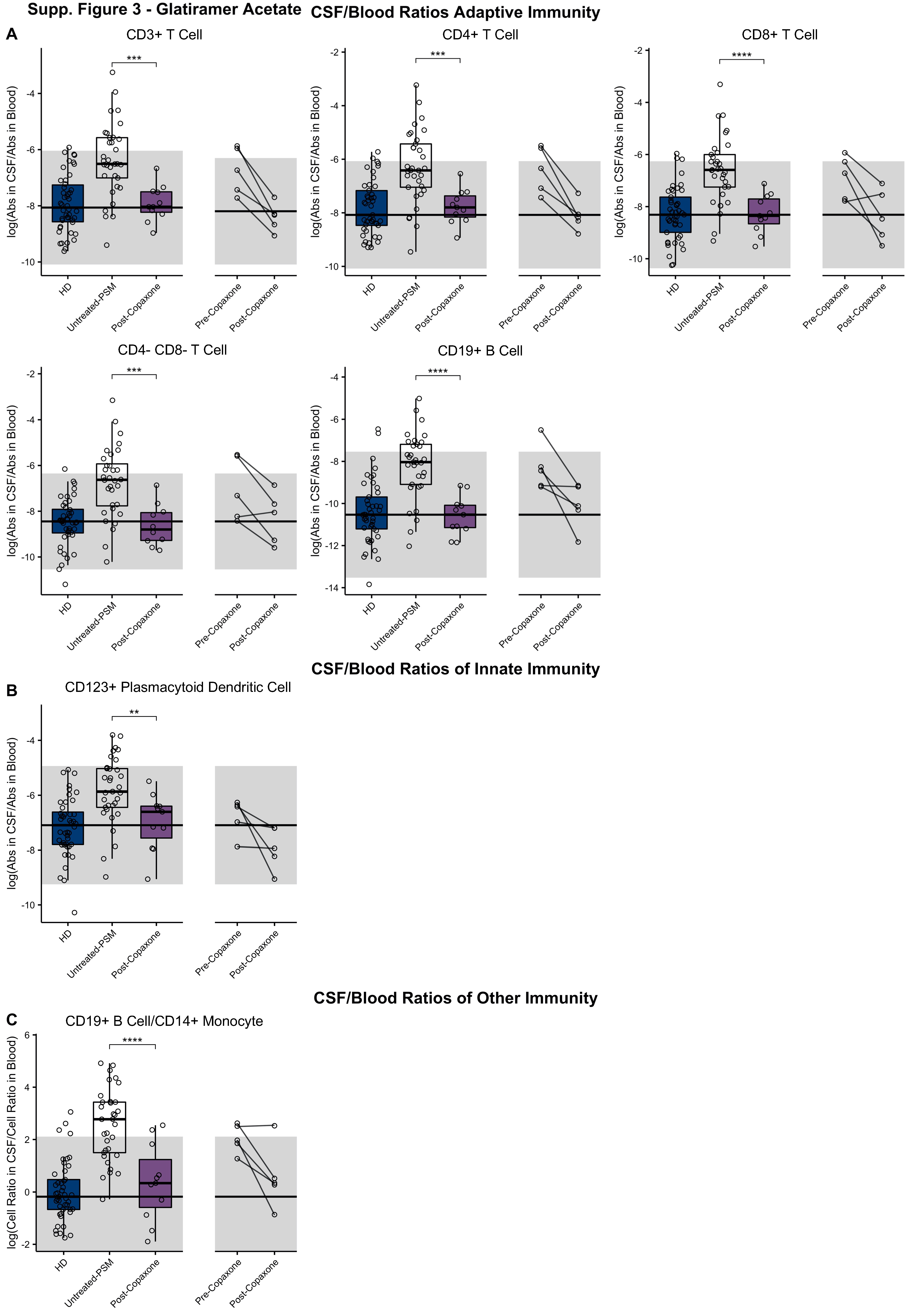

Supplement: Supplementary Figure 3 — Features that were statistically significant in blood and/or CSF between PSM untreated and GA-treated MS patients were calculated as CSF/blood ratios. CSF/blood ratios were then compared between 33 PSM untreated and 11 GA-treated MS patients and an unpaired t-test with adjustment for multiple comparisons performed. All significant markers were graphed and supplemented with HD cohort and data from 5 longitudinal patients with paired pretreatment and post-treatment data. *p < 0.05, **0.01 < p < 0.005, ***p < 0.001, ****p < 0.0001. The HD median for each feature was also graphed horizontally and gray shading added representing ± 2 SDs of each feature in the HD cohort. (A) Features in adaptive immunity. (B) Features in innate immunity. (C) Features in other immunity. [file Image_3.tif]

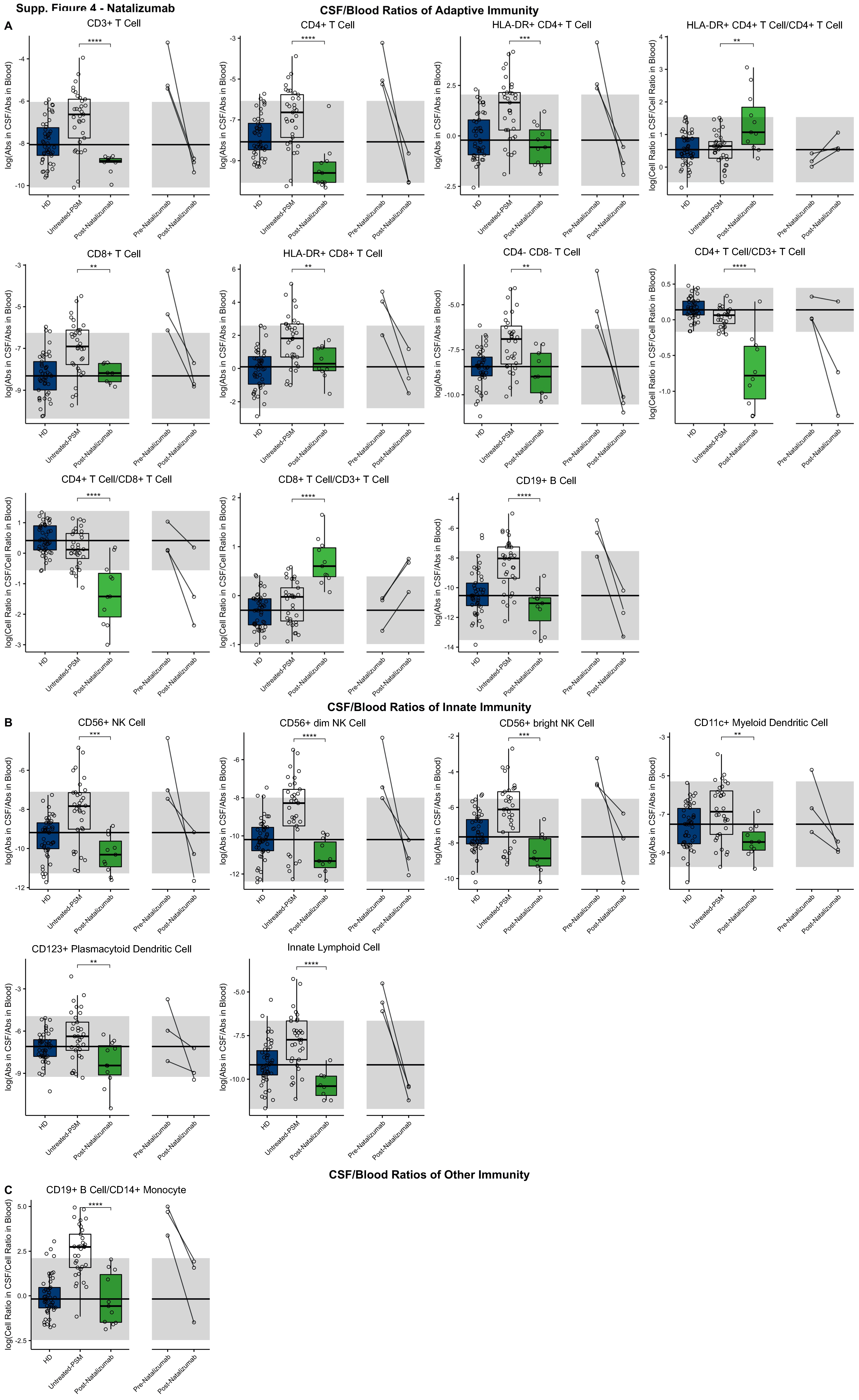

Supplement: Supplementary Figure 4 — Features that were statistically significant in blood and/or CSF between PSM untreated and natalizumab-treated MS patients were calculated as CSF/blood ratios. CSF/blood ratios were then compared between 33 PSM untreated and 11 natalizumab-treated MS patients and an unpaired t-test with adjustment for multiple comparisons performed. All significant markers were graphed and supplemented with HD cohort and data from 3 longitudinal patients with paired pretreatment and post-treatment data. *p < 0.05, **0.01 < p < 0.005, ***p < 0.001, ****p < 0.0001. The HD median for each feature was also graphed horizontally and gray shading added representing ± 2 SDs of each feature in the HD cohort. (A) Features in adaptive immunity. (B) Features in innate immunity. (C) Features in other immunity. [file Image_4.tif]

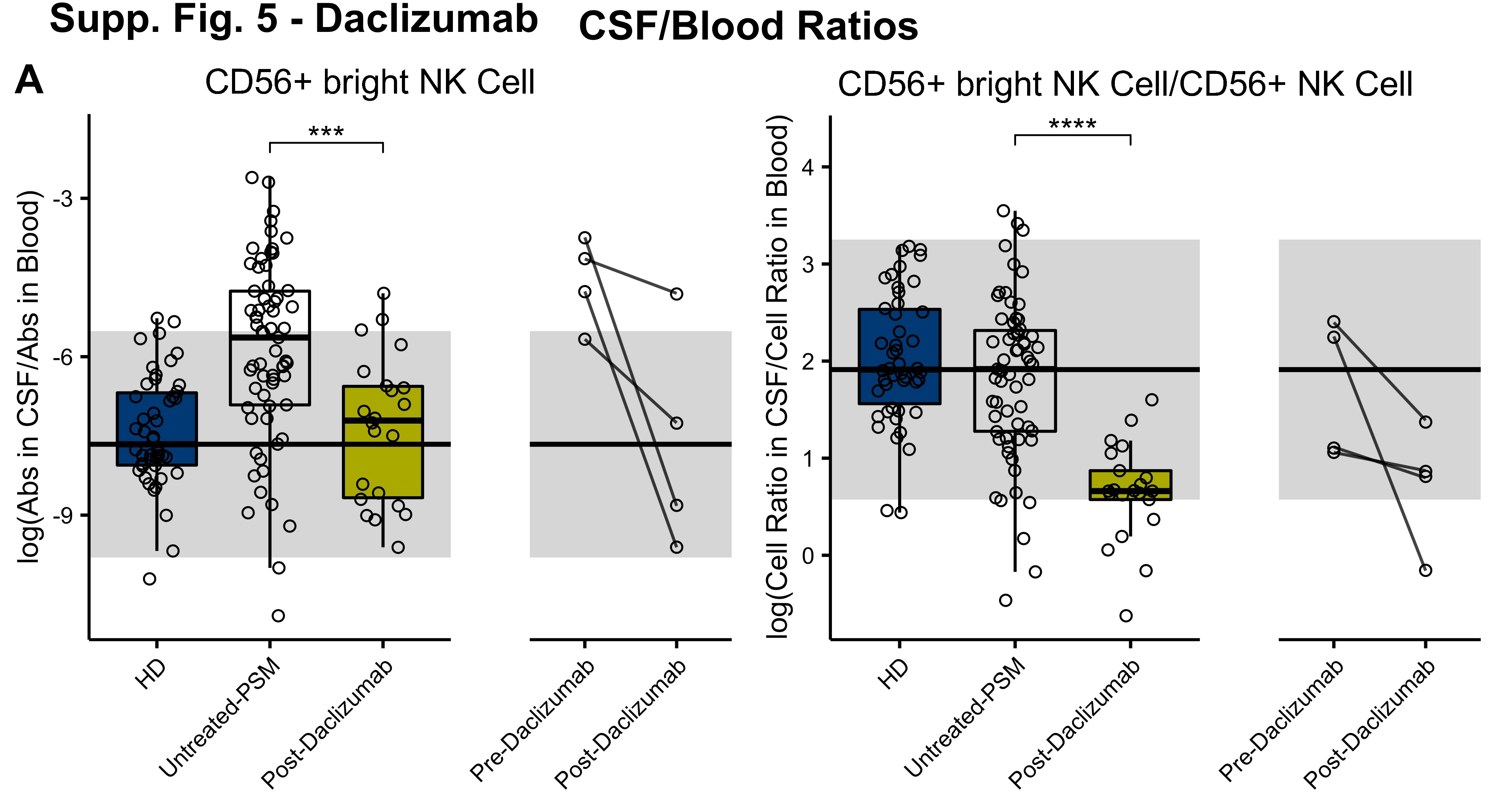

Supplement: Supplementary Figure 5 — Features that were statistically significant in blood and/or CSF between PSM untreated and daclizumab-treated MS patients were calculated as CSF/blood ratios. CSF/blood ratios were then compared between 66 PSM untreated and 22 daclizumab-treated MS patients and an unpaired t-test with adjustment for multiple comparisons performed. All significant markers were graphed and supplemented with HD cohort and data from 4 longitudinal patients with paired pretreatment and post-treatment data. *p < 0.05, **0.01 < p < 0.005, ***p < 0.001, ****p < 0.0001. The HD median for each feature was also graphed horizontally and gray shading added representing ± 2 SDs of each feature in the HD cohort. (A) Features in innate immunity. [file Image_5.tif]

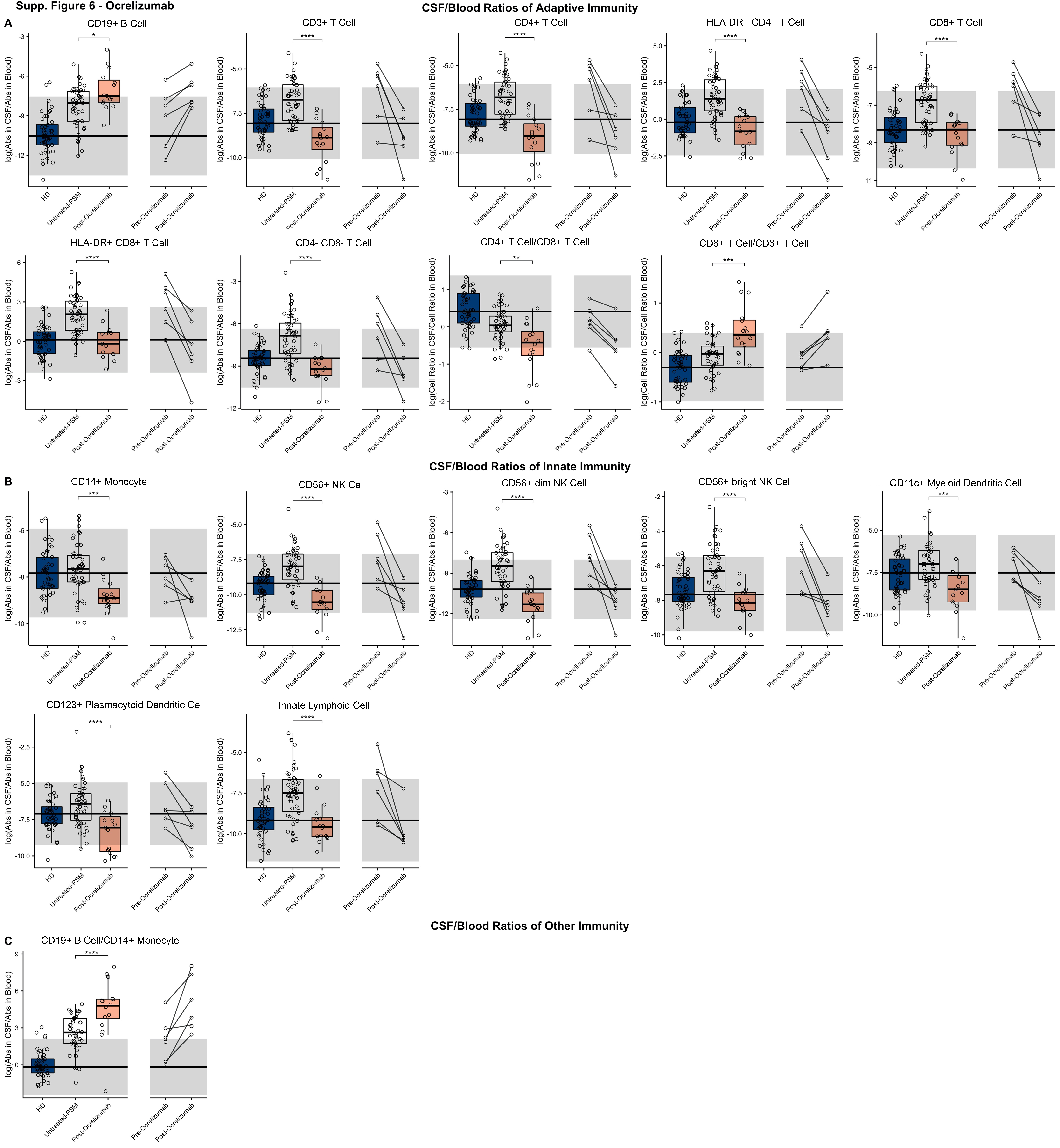

Supplement: Supplementary Figure 6 — Features that were statistically significant in blood and/or CSF between PSM untreated and ocrelizumab-treated MS patients were calculated as CSF/blood ratios. CSF/blood ratios were then compared between 48 PSM untreated and 16 ocrelizumab-treated MS patients and an unpaired t-test with adjustment for multiple comparisons performed. All significant markers were graphed and supplemented with HD cohort and data from 6 longitudinal patients with paired pretreatment and post-treatment data. *p < 0.05, **0.01 < p < 0.005, ***p < 0.001, ****p < 0.0001. The HD median for each feature was also graphed horizontally and gray shading added representing ± 2 SDs of each feature in HD cohort. (A) Features in adaptive immunity. (B) Features in innate immunity. (C) Features in other immunity. [file Image_6.tiff]
