# Supplementary material for: Extensive Healthy Donor Age/Gender Adjustments and Propensity Score Matching Reveal Physiology of Multiple Sclerosis Through Immunophenotyping
Source: Front Neurol. 2020 Nov 27;11:565957. doi: 10.3389/fneur.2020.565957 (PMC7732581; doi:10.3389/fneur.2020.565957)
Supplement: Supplementary Presentation 1 — Major results of this study are described in this presentation, beginning with a description of immune cell populations in healthy blood and CSF and further narration of age and gender effects on these populations in HDs. Then RRMS and PMS patient blood and CSF immune system physiologies are differentiated from HDs. Last, IFN-beta, GA, natalizumab, daclizumab, and ocrelizumab treated MS patients are compared to untreated MS patients in this 15-min video. [file Presentation_1.pdf]

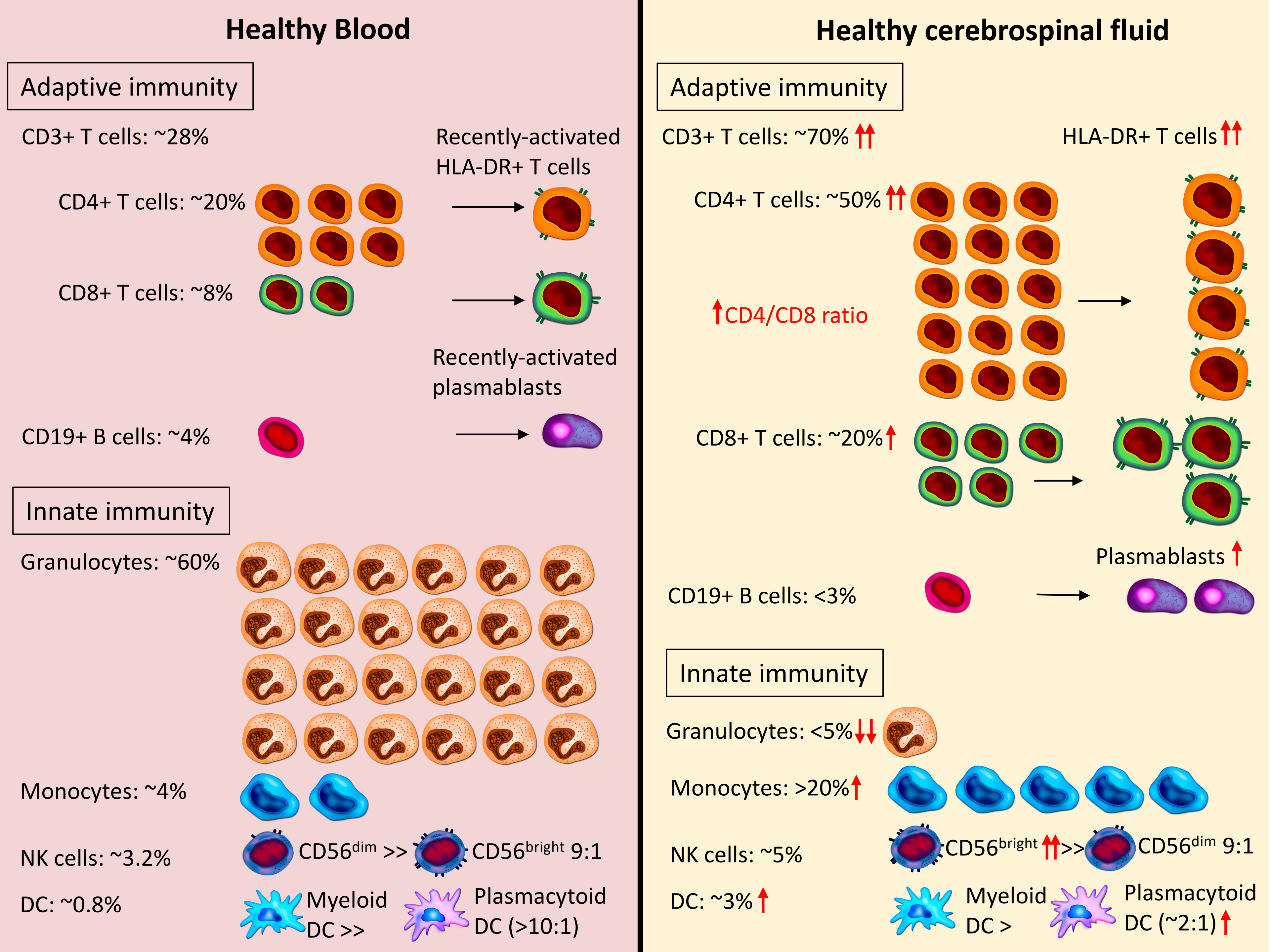
Healthy Blood
Adaptive immunity
CD3+ T cells: ~28%
Recently-activated HLA-DR+ T cells
CD4+ T cells: ~20%
CD8+ T cells: ~8%
Recently-activated plasmablasts
CD19+ B cells: ~4%
Innate immunity
Granulocytes: ~60%
Monocytes: ~4%
NK cells: ~3.2%
CD56dim >> CD56bright 9:1
DC: ~0.8%
Myeloid DC >>
Plasmacytoid DC (>10:1)
Healthy cerebrospinal fluid
Adaptive immunity
CD3+ T cells: ~70%
HLA-DR+ T cells
CD4+ T cells: ~50%
CD4/CD8 ratio
CD8+ T cells: ~20%
Plasmablasts
CD19+ B cells: <3%
Innate immunity
Granulocytes: <5%
Monocytes: >20%
NK cells: ~5%
CD56bright >> CD56dim 9:1
DC: ~3%
Myeloid DC >
Plasmacytoid DC (~2:1)

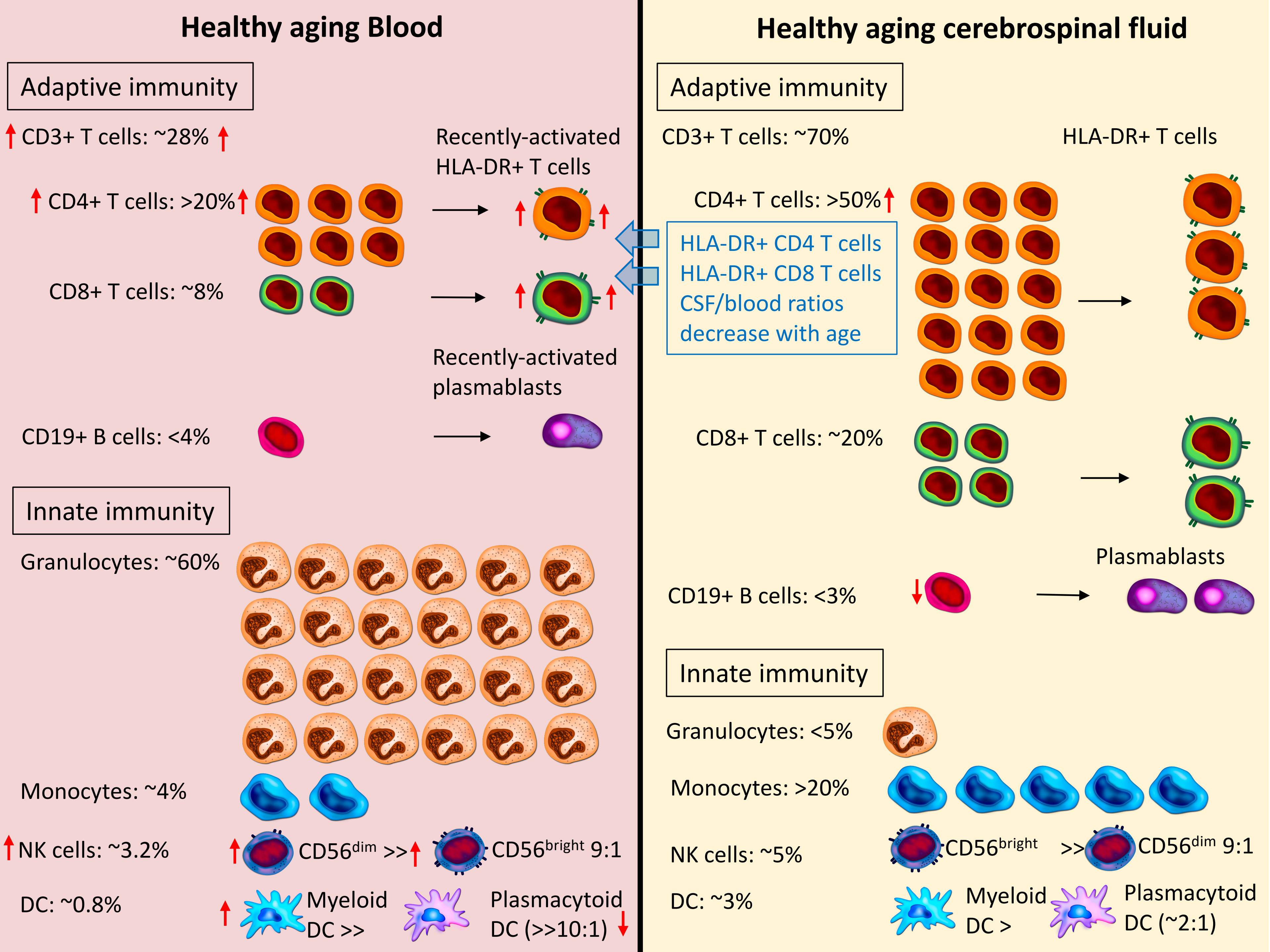

Healthy aging Blood
Adaptive immunity
CD3+ T cells: ~28%
Recently-activated HLA-DR+ T cells
CD4+ T cells: >20%
CD8+ T cells: ~8%
Recently-activated plasmablasts
CD19+ B cells: <4%
Innate immunity
Granulocytes: ~60%
Monocytes: ~4%
NK cells: ~3.2%
CD56dim >> CD56bright 9:1
DC: ~0.8%
Myeloid DC >> Plasmacytoid DC (>>10:1)
Healthy aging cerebrospinal fluid
Adaptive immunity
CD3+ T cells: ~70%
HLA-DR+ T cells
CD4+ T cells: >50%
HLA-DR+ CD4 T cells
HLA-DR+ CD8 T cells
CSF/blood ratios
decrease with age
CD8+ T cells: ~20%
Plasmablasts
CD19+ B cells: <3%
Innate immunity
Granulocytes: <5%
Monocytes: >20%
NK cells: ~5%
CD56bright >> CD56dim 9:1
DC: ~3%
Myeloid DC > Plasmacytoid DC (~2:1)

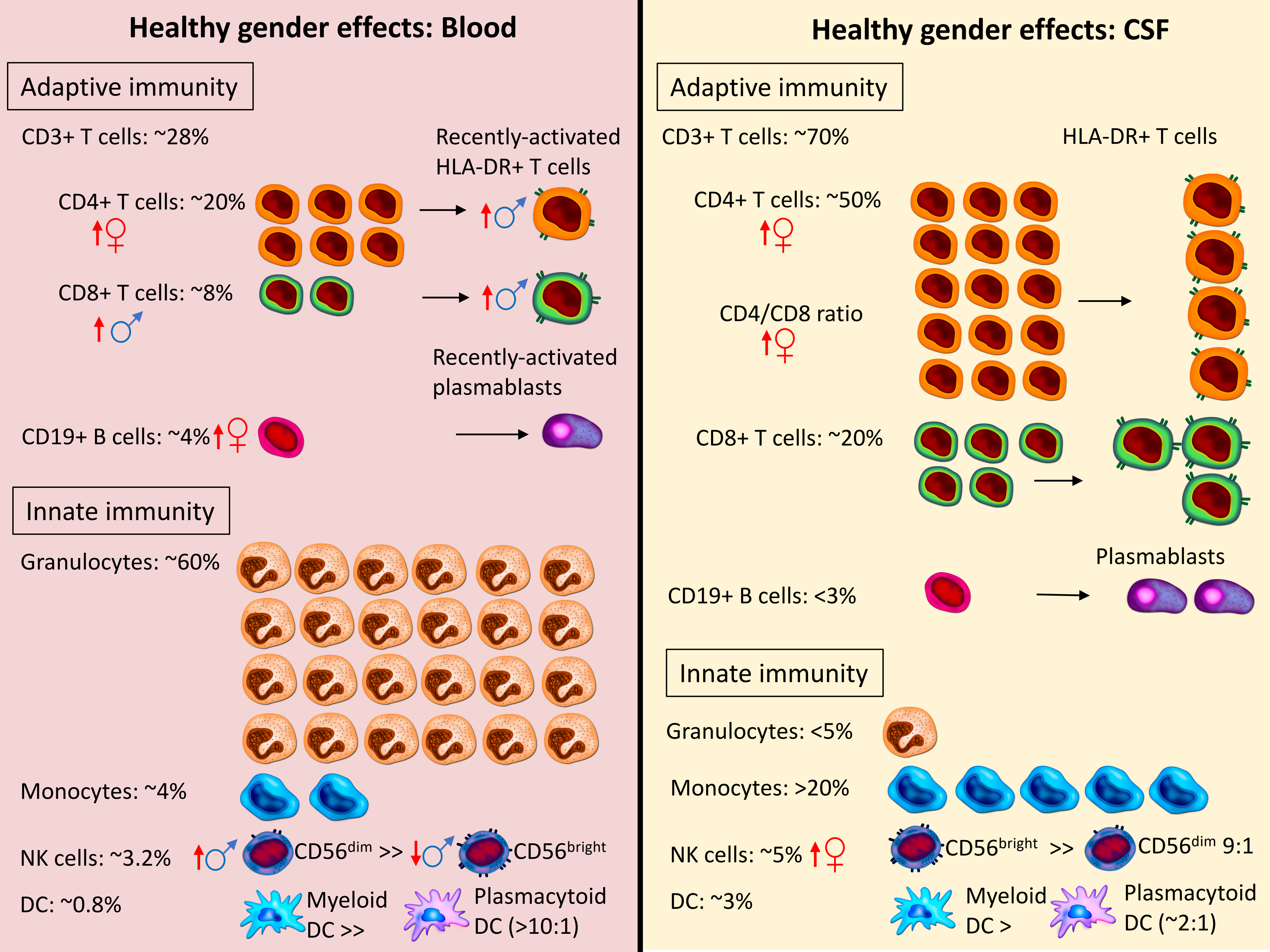
Healthy gender effects: Blood
Adaptive immunity
CD3+ T cells: ~28%
Recently-activated HLA-DR+ T cells
CD4+ T cells: ~20% ↑♀
↑♂
CD8+ T cells: ~8% ↑♂
↑♂
Recently-activated plasmablasts
CD19+ B cells: ~4% ↑♀
Innate immunity
Granulocytes: ~60%
Monocytes: ~4%
NK cells: ~3.2% ↑♂ CD56dim >> ↓♂ CD56bright
DC: ~0.8%
Myeloid DC >> Plasmacytoid DC (>10:1)
Healthy gender effects: CSF
Adaptive immunity
CD3+ T cells: ~70%
HLA-DR+ T cells
CD4+ T cells: ~50% ↑♀
CD4/CD8 ratio ↑♀
CD8+ T cells: ~20%
Plasmablasts
CD19+ B cells: <3%
Innate immunity
Granulocytes: <5%
Monocytes: >20%
NK cells: ~5% ↑♀
CD56bright >> CD56dim 9:1
DC: ~3%
Myeloid DC > Plasmacytoid DC (~2:1)

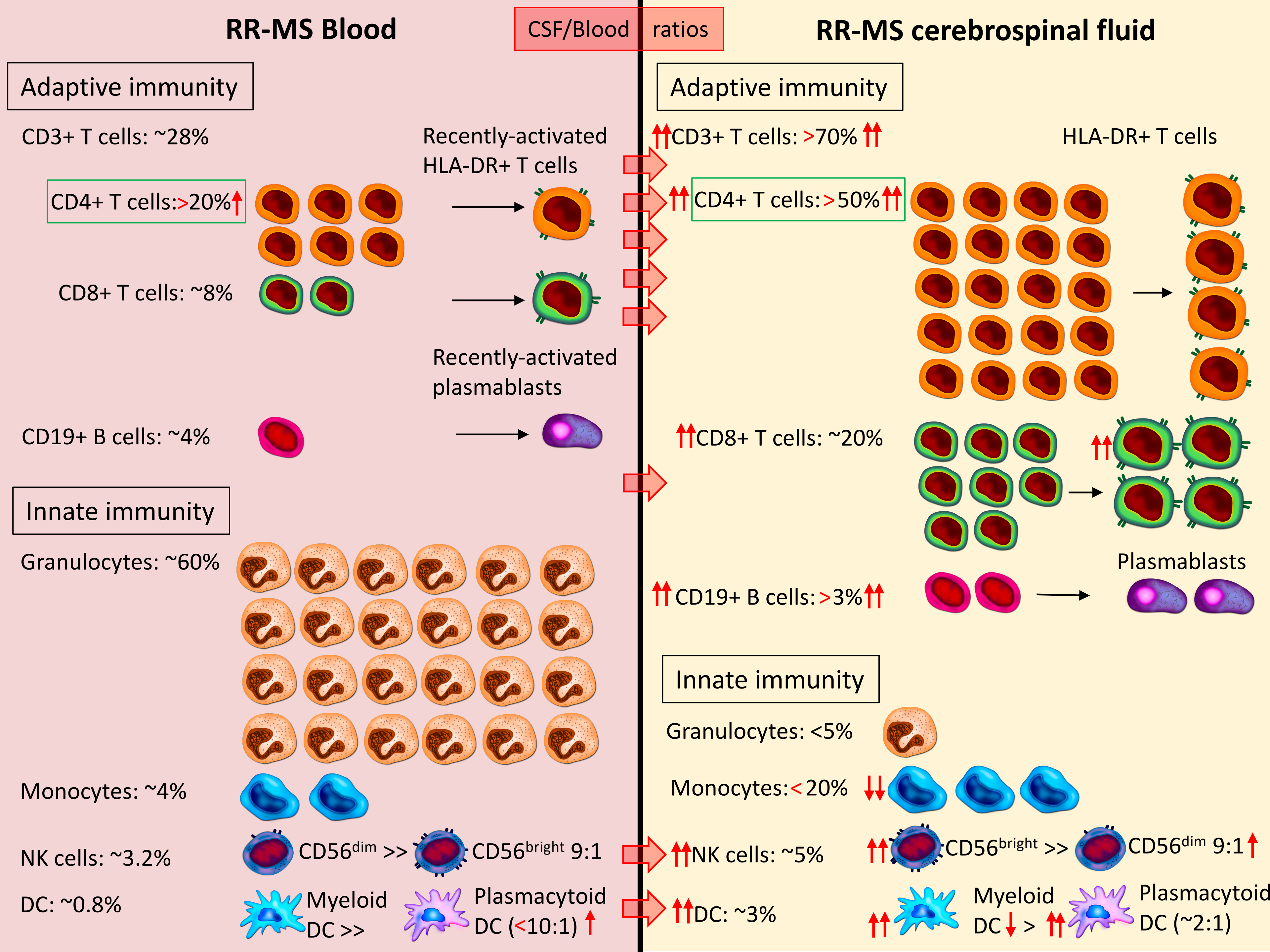
RR-MS Blood
CSF/Blood ratios
RR-MS cerebrospinal fluid
Adaptive immunity
CD3+ T cells: ~28%
Recently-activated HLA-DR+ T cells
CD4+ T cells: >20%
CD8+ T cells: ~8%
Recently-activated plasmablasts
CD19+ B cells: ~4%
Innate immunity
Granulocytes: ~60%
Monocytes: ~4%
NK cells: ~3.2%
CD56dim >> CD56bright 9:1
DC: ~0.8%
Myeloid DC >> Plasmacytoid DC (<10:1)
Adaptive immunity
CD3+ T cells: >70%
HLA-DR+ T cells
CD4+ T cells: >50%
CD8+ T cells: ~20%
Plasmablasts
CD19+ B cells: >3%
Innate immunity
Granulocytes: <5%
Monocytes: < 20%
NK cells: ~5%
CD56bright >> CD56dim 9:1
DC: ~3%
Myeloid DC > Plasmacytoid DC (~2:1)

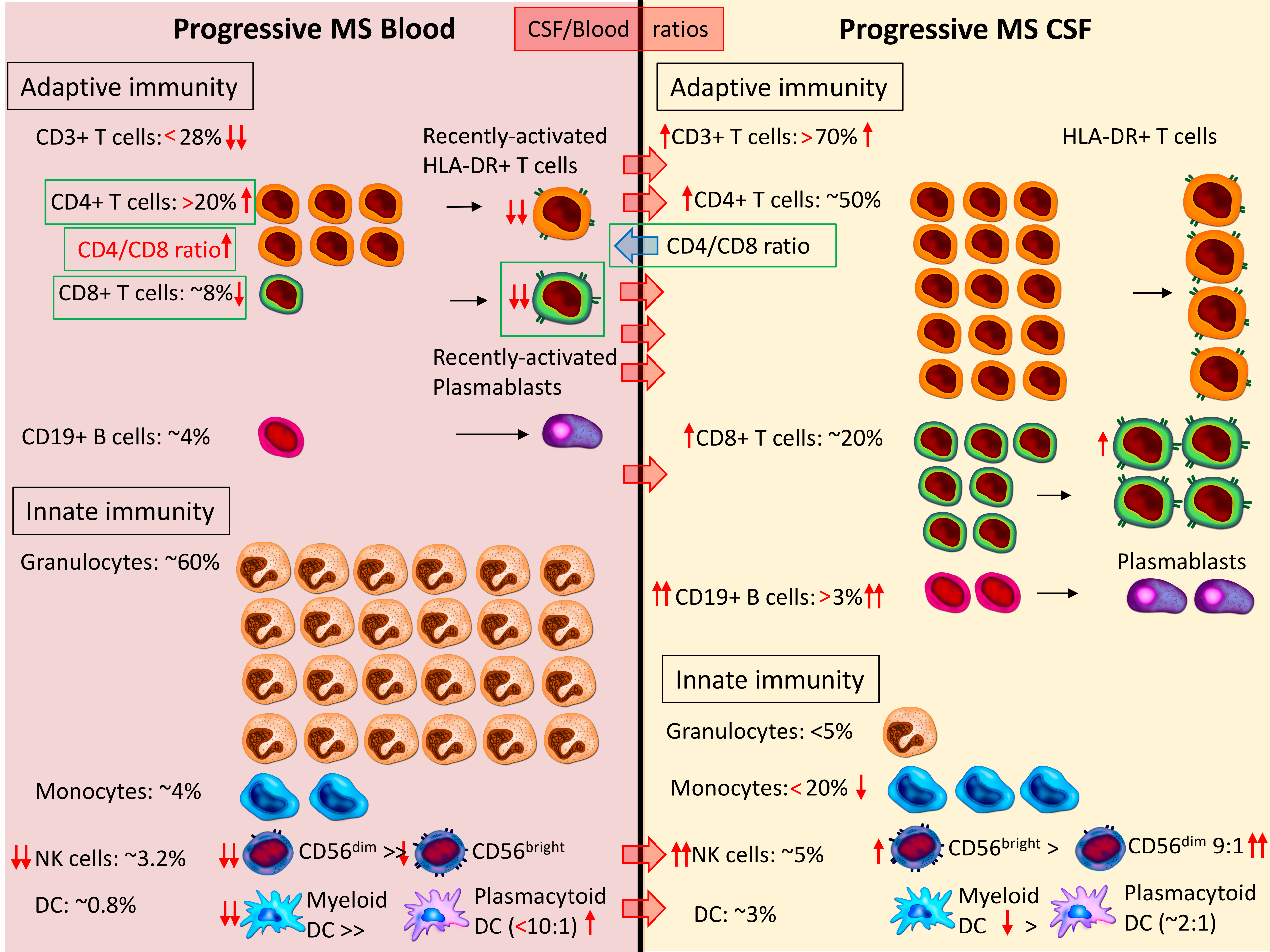

Progressive MS Blood
CSF/Blood ratios
Progressive MS CSF
Adaptive immunity
CD3+ T cells: <28%
Recently-activated
HLA-DR+ T cells
CD4+ T cells: >20%
CD4/CD8 ratio
CD8+ T cells: ~8%
Recently-activated
Plasmablasts
CD19+ B cells: ~4%
Innate immunity
Granulocytes: ~60%
Monocytes: ~4%
NK cells: ~3.2%
CD56dim >> CD56bright
DC: ~0.8%
Myeloid DC >> Plasmacytoid DC (<10:1)
Adaptive immunity
CD3+ T cells: >70%
HLA-DR+ T cells
CD4+ T cells: ~50%
CD4/CD8 ratio
CD8+ T cells: ~20%
Plasmablasts
CD19+ B cells: >3%
Innate immunity
Granulocytes: <5%
Monocytes: < 20%
NK cells: ~5%
CD56bright > CD56dim 9:1
DC: ~3%
Myeloid DC > Plasmacytoid DC (~2:1)

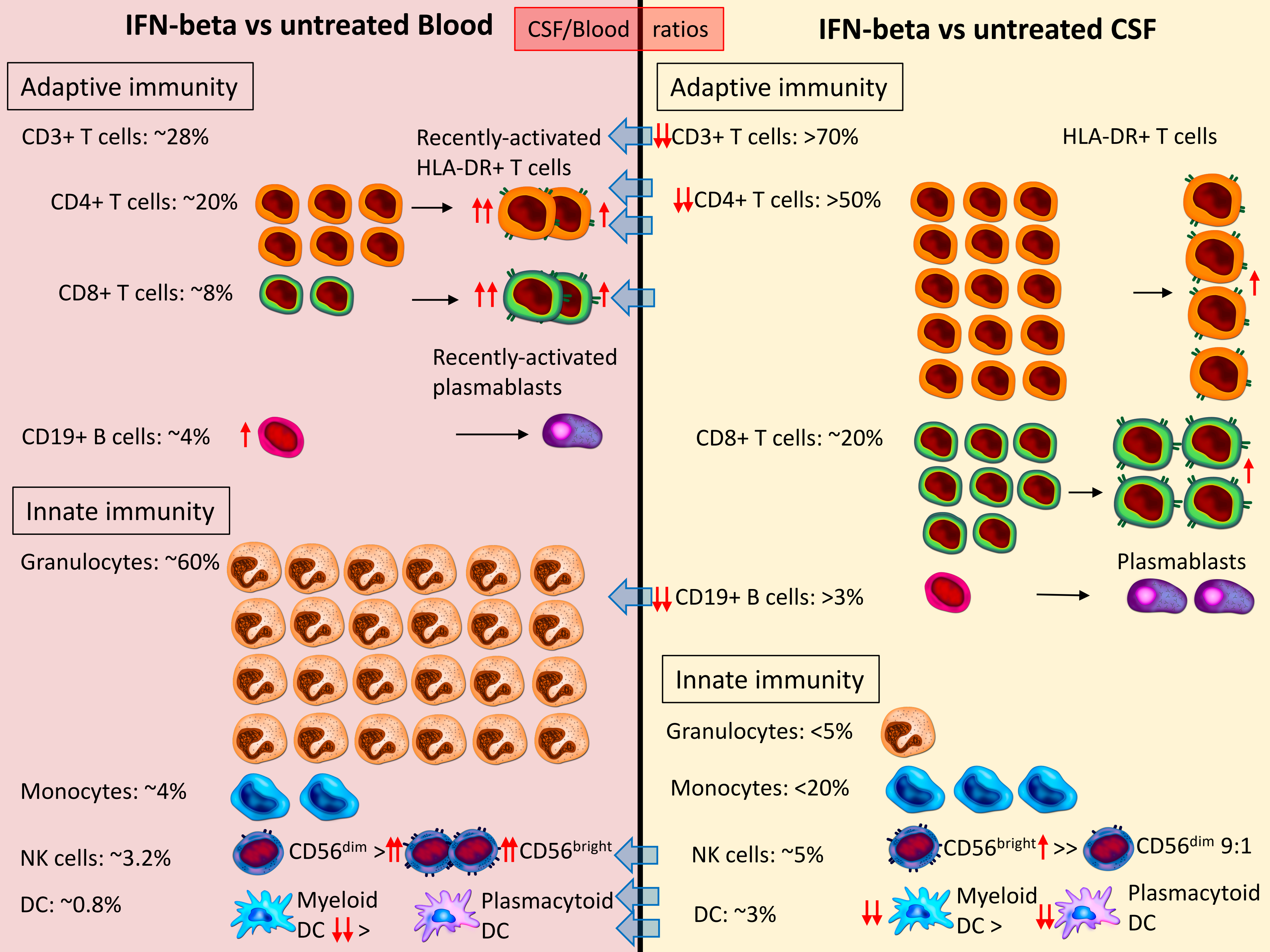

IFN-beta vs untreated Blood
CSF/Blood ratios
IFN-beta vs untreated CSF
Adaptive immunity
CD3+ T cells: ~28%
Recently-activated HLA-DR+ T cells
CD4+ T cells: ~20%
CD8+ T cells: ~8%
Recently-activated plasmablasts
CD19+ B cells: ~4%
Innate immunity
Granulocytes: ~60%
Monocytes: ~4%
NK cells: ~3.2%
CD56dim > CD56bright
DC: ~0.8%
Myeloid DC > Plasmacytoid DC
Adaptive immunity
CD3+ T cells: >70%
HLA-DR+ T cells
CD4+ T cells: >50%
CD8+ T cells: ~20%
Plasmablasts
CD19+ B cells: >3%
Innate immunity
Granulocytes: <5%
Monocytes: <20%
NK cells: ~5%
CD56bright >> CD56dim 9:1
DC: ~3%
Myeloid DC > Plasmacytoid DC

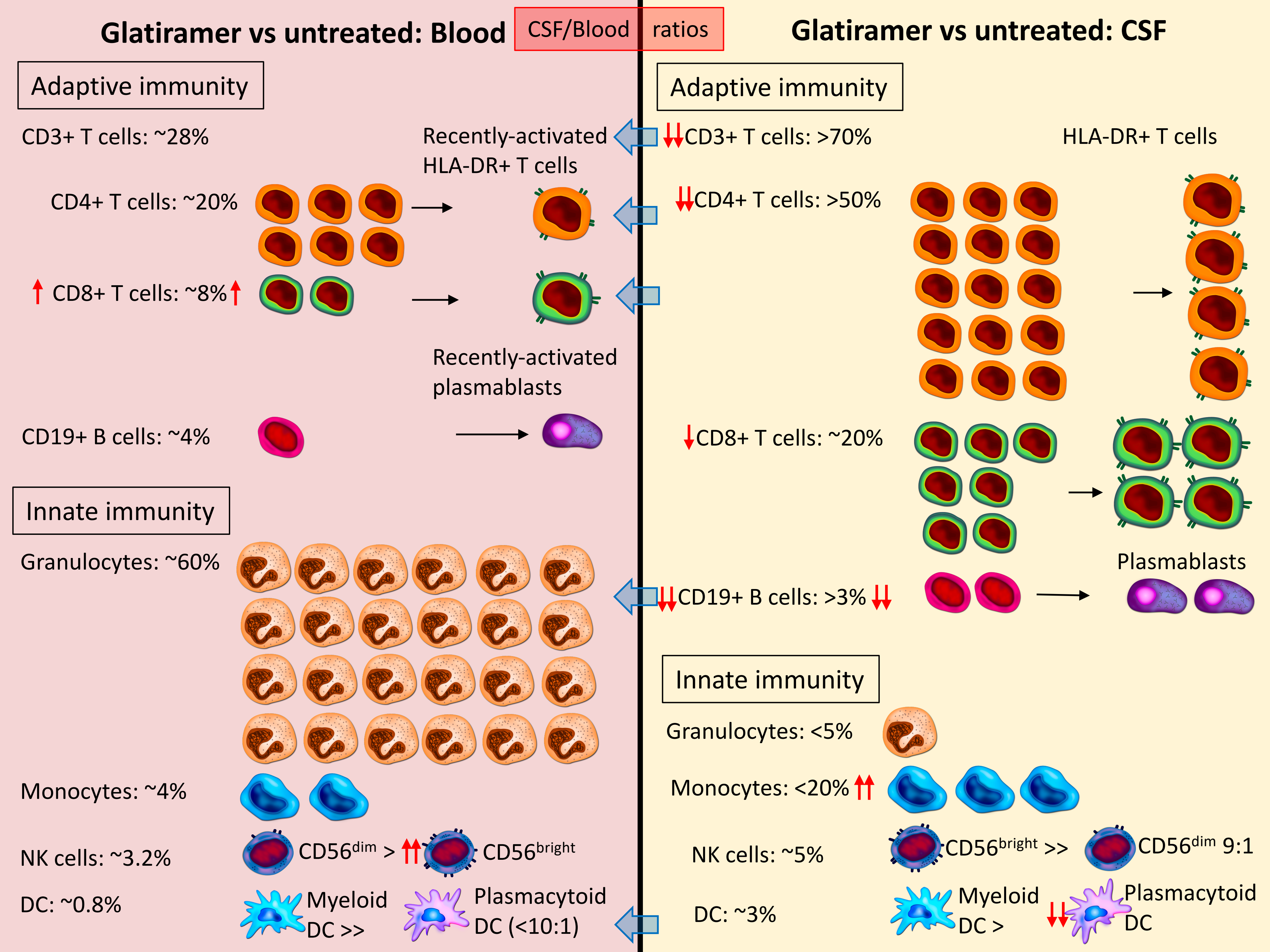

Glatiramer vs untreated: Blood
CSF/Blood ratios
Glatiramer vs untreated: CSF
Adaptive immunity
CD3+ T cells: ~28%
Recently-activated HLA-DR+ T cells
CD4+ T cells: ~20%
CD8+ T cells: ~8%
Recently-activated plasmablasts
CD19+ B cells: ~4%
Innate immunity
Granulocytes: ~60%
Monocytes: ~4%
NK cells: ~3.2%
CD56dim > CD56bright
DC: ~0.8%
Myeloid DC >>
Plasmacytoid DC (<10:1)
Adaptive immunity
CD3+ T cells: >70%
HLA-DR+ T cells
CD4+ T cells: >50%
CD8+ T cells: ~20%
Plasmablasts
CD19+ B cells: >3%
Innate immunity
Granulocytes: <5%
Monocytes: <20%
NK cells: ~5%
CD56bright >> CD56dim 9:1
DC: ~3%
Myeloid DC >
Plasmacytoid DC

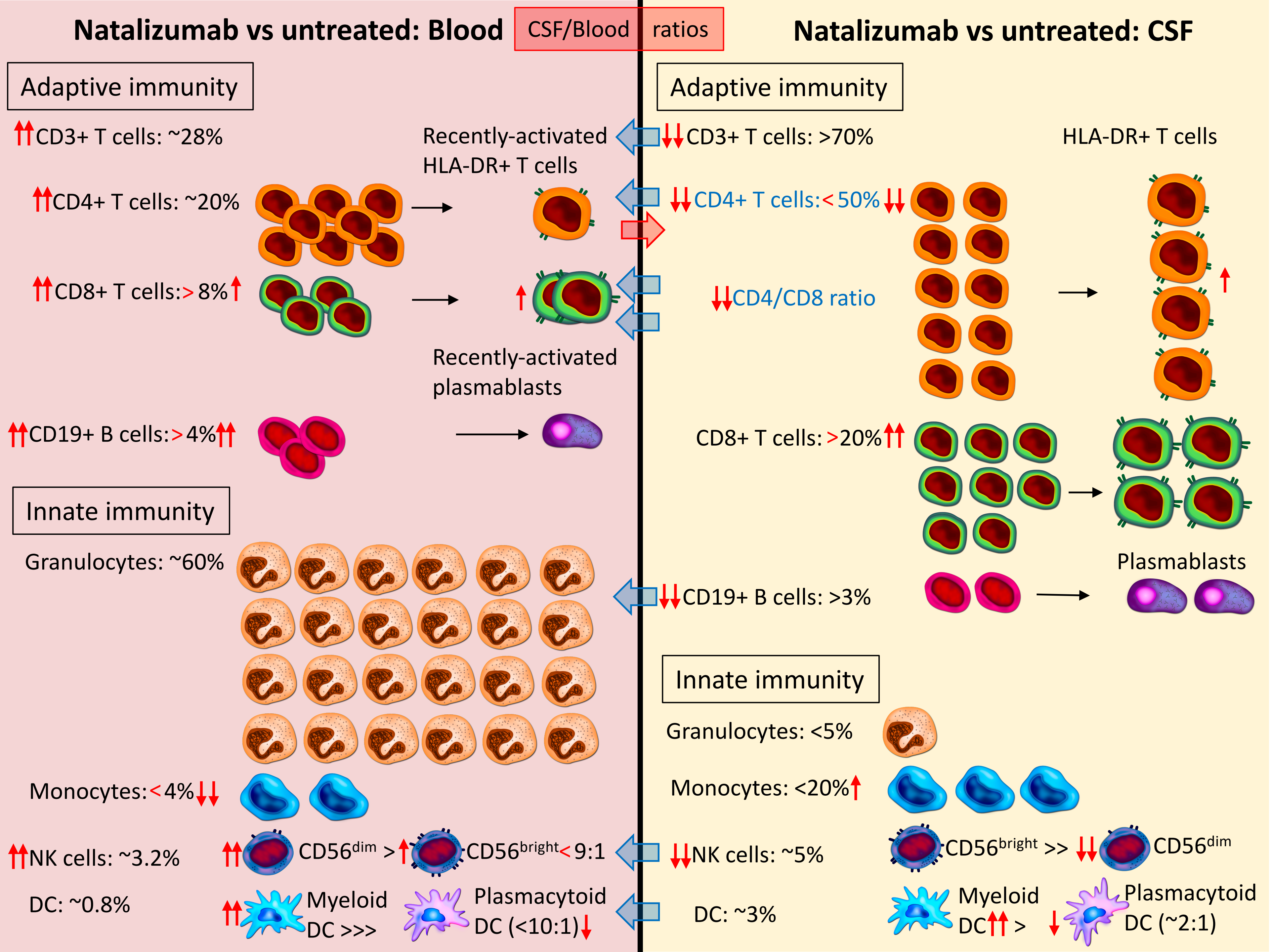

Natalizumab vs untreated: Blood
CSF/Blood ratios
Natalizumab vs untreated: CSF
Adaptive immunity
CD3+ T cells: ~28%
Recently-activated HLA-DR+ T cells
CD4+ T cells: ~20%
CD8+ T cells: > 8%
Recently-activated plasmablasts
CD19+ B cells: > 4%
Innate immunity
Granulocytes: ~60%
Monocytes: < 4%
NK cells: ~3.2%
CD56^{dim} > CD56^{bright} < 9:1
DC: ~0.8%
Myeloid DC >>>
Plasmacytoid DC (<10:1)
Adaptive immunity
CD3+ T cells: >70%
HLA-DR+ T cells
CD4+ T cells: < 50%
CD4/CD8 ratio
CD8+ T cells: > 20%
Plasmablasts
CD19+ B cells: >3%
Innate immunity
Granulocytes: <5%
Monocytes: <20%
NK cells: ~5%
CD56^{bright} >> CD56^{dim}
DC: ~3%
Myeloid DC >
Plasmacytoid DC (~2:1)

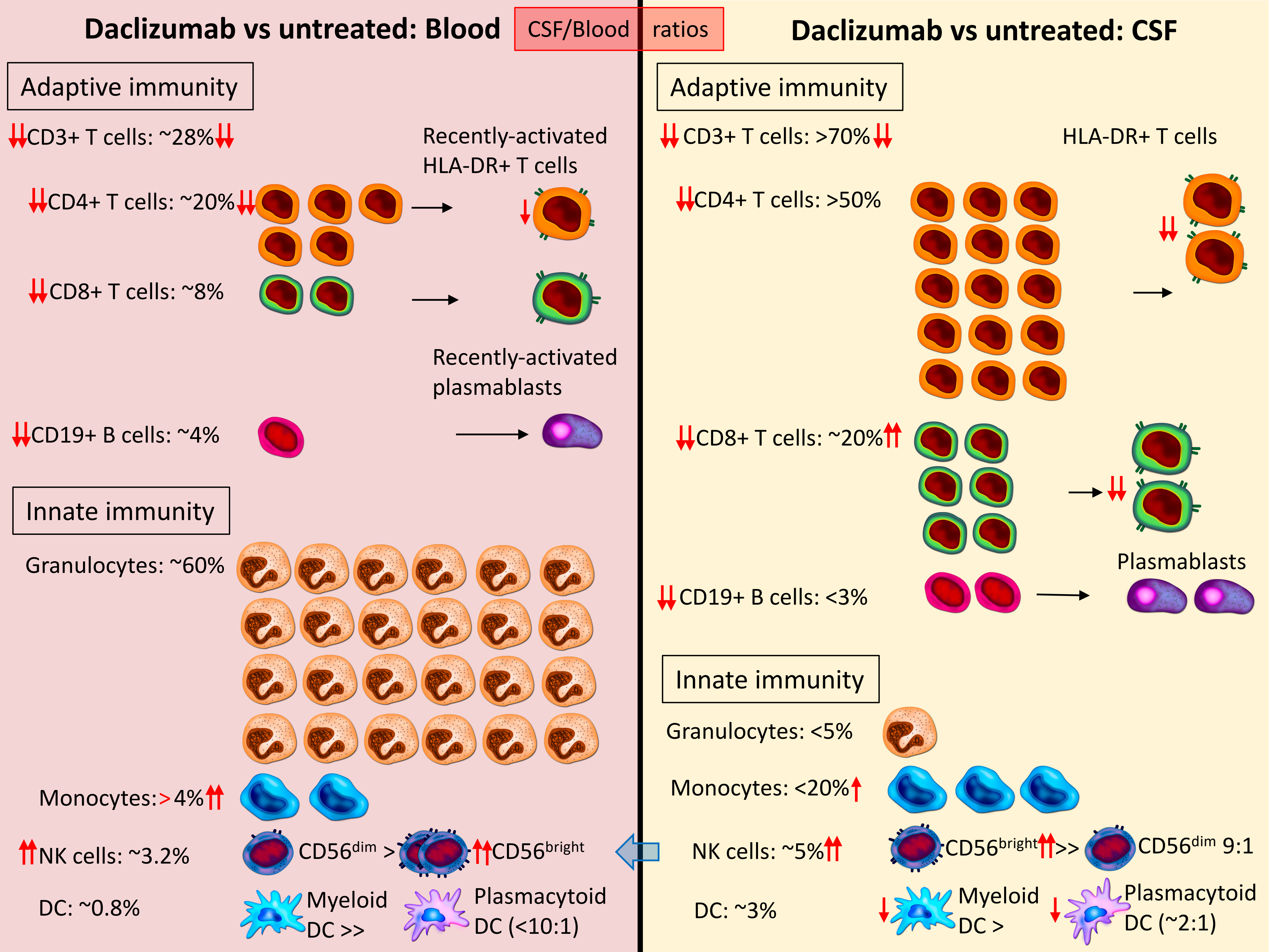
Daclizumab vs untreated: Blood
CSF/Blood ratios
Daclizumab vs untreated: CSF
Adaptive immunity
CD3+ T cells: ~28%
Recently-activated HLA-DR+ T cells
CD4+ T cells: ~20%
CD8+ T cells: ~8%
Recently-activated plasmablasts
CD19+ B cells: ~4%
Innate immunity
Granulocytes: ~60%
Monocytes: >4%
NK cells: ~3.2%
CD56^{dim} > CD56^{bright}
DC: ~0.8%
Myeloid DC >>
Plasmacytoid DC (<10:1)
Adaptive immunity
CD3+ T cells: >70%
HLA-DR+ T cells
CD4+ T cells: >50%
CD8+ T cells: ~20%
Plasmablasts
CD19+ B cells: <3%
Innate immunity
Granulocytes: <5%
Monocytes: <20%
NK cells: ~5%
CD56^{bright} >> CD56^{dim} 9:1
DC: ~3%
Myeloid DC >
Plasmacytoid DC (~2:1)

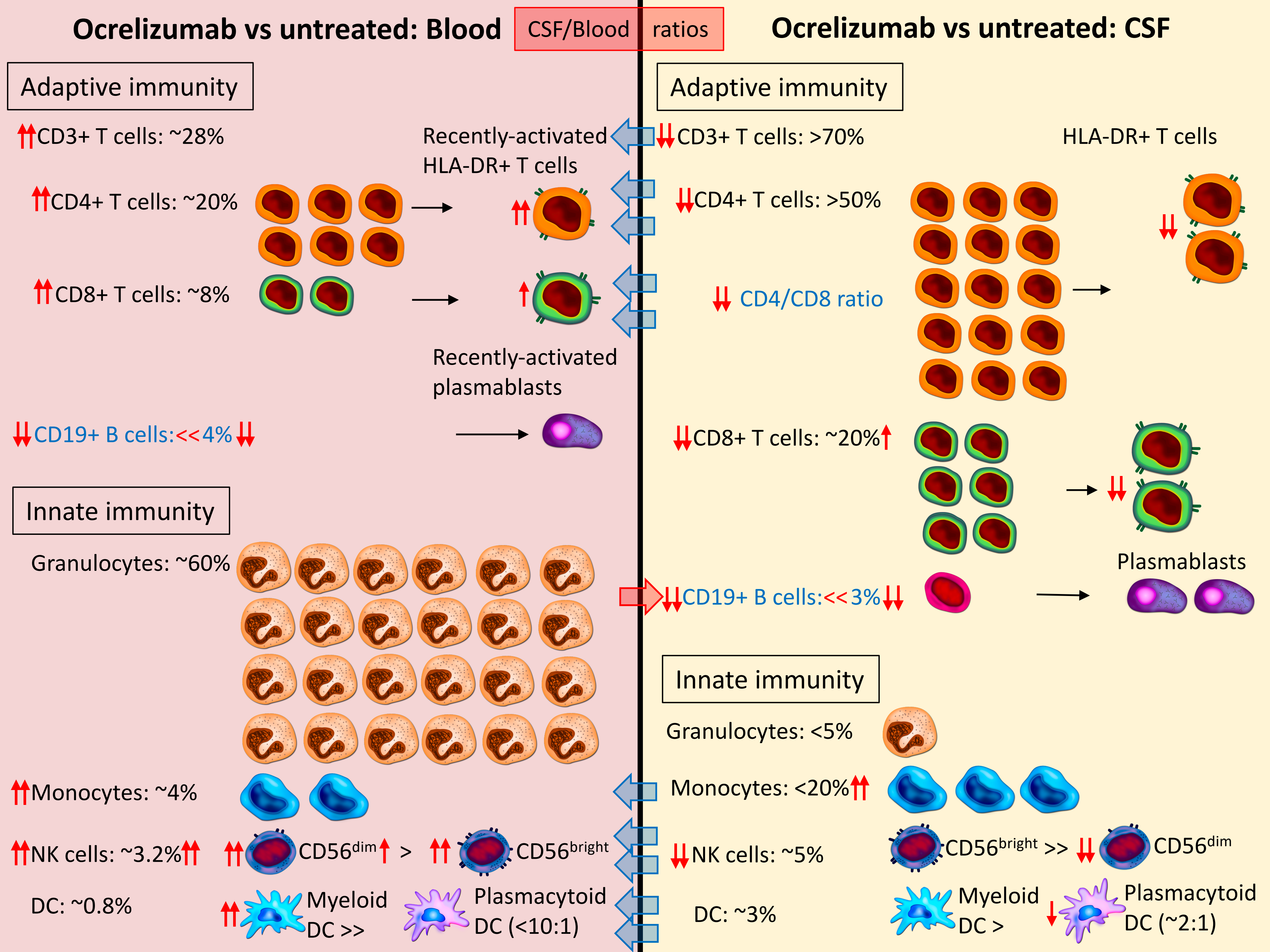
Ocrelizumab vs untreated: Blood
CSF/Blood ratios
Ocrelizumab vs untreated: CSF
Adaptive immunity
CD3+ T cells: ~28%
Recently-activated HLA-DR+ T cells
CD4+ T cells: ~20%
CD8+ T cells: ~8%
Recently-activated plasmablasts
CD19+ B cells:<<4%
Innate immunity
Granulocytes: ~60%
Monocytes: ~4%
NK cells: ~3.2%
CD56dim > CD56bright
DC: ~0.8%
Myeloid DC >> Plasmacytoid DC (<10:1)
Adaptive immunity
CD3+ T cells: >70%
HLA-DR+ T cells
CD4+ T cells: >50%
CD4/CD8 ratio
CD8+ T cells: ~20%
Plasmablasts
CD19+ B cells:<<3%
Innate immunity
Granulocytes: <5%
Monocytes: <20%
NK cells: ~5%
CD56bright >> CD56dim
DC: ~3%
Myeloid DC > Plasmacytoid DC (~2:1)
